# Complexation of Cyclodextrins with Benzoic Acid in Water-Organic Solvents: A Solvation-Thermodynamic Approach

**DOI:** 10.3390/molecules26154408

**Published:** 2021-07-21

**Authors:** Tatyana R. Usacheva, Vitaly A. Volynkin, Viktor T. Panyushkin, Dmitry A. Lindt, Thi Lan Pham, Thi Thu Ha Nguyen, Thi My Hanh Le, Diana A. Alister, Dzhovidon N. Kabirov, Natalya N. Kuranova, George A. Gamov, Roman A. Kushnir, Marco Biondi, Concetta Giancola, Valentin A. Sharnin

**Affiliations:** 1 Department of Inorganic Chemistry and Technology, Ivanovo State University of Chemistry and Technology, 153000 Ivanovo, Russia; matchoaa@mail.ru (D.A.A.); kabirov93.93@mail.ru (D.N.K.); kuranova_nn@isuct.ru (N.N.K.); ggamov@isuct.ru (G.A.G.); oxt705@isuct.ru (R.A.K.); sharn@isuct.ru (V.A.S.); 2 Department of Chemistry and High Technologies, Kuban State University, 350040 Krasnodar, Russia; vva@chem.kubsu.ru (V.A.V.); panyushkin@chem.kubsu.ru (V.T.P.); chem.lindt_d.a@mail.ru (D.A.L.); 3 Laboratory of Applied Research and Technology, Institute for Tropical Technology, Vietnam Academy of Science and Technology, Hanoi 100000, Vietnam; ptlan@itt.vast.vn (T.L.P.); hanhltm76@gmail.com (T.M.H.L.); 4 Faculty of Chemistry, Hanoi National University of Education, Hanoi 100000, Vietnam; ntt.ha@hnue.edu.vn; 5 Department of Pharmaceutical and Toxicological Chemistry, Avicenna Tajik State Medical University, Dushanbe 734003, Tajikistan; 6 Department of Pharmacy, University of Naples Federico II, 80131 Naples, Italy; mabiondi@unina.it (M.B.); giancola@unina.it (C.G.)

**Keywords:** benzoic acid, binary solvents, β-cyclodextrin, quantum chemical calculations, inclusion complexes, NMR spectroscopy, stability constant, solvation-thermodynamic approach, thermodynamic parameters

## Abstract

The aim of this research is to obtain new data about the complexation between β-cyclodextrin (β-CD) and benzoic acid (BA) as a model reaction of the complex formation of hydrophobic molecules with cyclodextrins (CDs) in various media. This research may help developing cyclodextrin-based pharmaceutical formulations through the choice of the appropriate solvent mixture that may be employed in the industrial application aiming to control the reactions/processes in liquid phase. In this paper, NMR results for the molecular complex formation between BA and β-CD ([BA⊂β-CD]) in D_2_O-DMSO-*d*_6_ and in D_2_O-EtOH have shown that the stability of the complex in the H_2_O-DMSO-*d*_6_ varies within the experimental error, while decreases in H_2_O-EtOH. Changes in the Gibbs energy of BA resolvation in water and water–dimethylsulfoxide mixtures have been obtained and have been used in the analysis of the reagent solvation contributions into the Gibbs energy changes of the [BA⊂β-CD] molecular complex formation. Quantum chemical calculations of the interaction energy between β-CD and BA as well as the structure of the [BA⊂β-CD] complex and the energy of β-CD and BA interaction in vacuum and in the medium of water, methanol and dimethylsulfoxide solvents are carried out. The stability of [BA⊂β-CD] complex in H_2_O-EtOH and H_2_O-DMSO solvents, obtained by different methods, are compared. The thermodynamic parameters of the [BA⊂β-CD] molecular complexation as well as the reagent solvation contributions in H_2_O-EtOH and H_2_O-DMSO mixtures were analyzed by the solvation-thermodynamic approach.

## 1. Introduction

Solvation plays a key role in all chemical and physico-chemical properties of solutions as well as virtually all processes occurring in solution. 

In the recent book *Scientific Schools of Ivanovo Chemtech: Through the Prism of History* [1], O.I. Koifman et al. pointed that it is necessary to significantly expand the experimental possibilities of thermodynamic and molecular-kinetic approaches to the study of solvate-thermodynamic effects in liquid systems. 

In recent studies [2,3], we have reported on the effect of reagents solvation on amine stability, the effect of reagents solvation on changes in the stability of amine, carboxylate, and coronate complexes of d-metal ions, as well as the thermodynamic characteristics of complexation reactions in water–organic solvents. General demonstrated trends in the changes of the thermodynamic characteristics of reactions allow us to predict modifications in the stability and the energy of coordination compounds that, in turn, depend on the solvation of reagents. Furthermore, the analysis of data on the thermodynamics of complexation reactions of the “metals of life” (e.g., silver (I), copper (II), iron (III), etc.) with selected members of class B vitamins (nicotinamide, nicotinic acid) allows for the isolation of the solvation contribution of the ligand reaction centers [4,5]. The use of results for binary mixtures of non-aqueous solvents allows to move away from water and its unique properties, thereby showing which solvation contributions determine the changes in the stability of the coordination compound and the energy of the reaction during the transition from one organic solvent to another [6,7]. The analysis of molecular complexation reactions of crown ethers and cryptands with amino acids and peptides shows that the solvation contributions of the host and guest molecules play a different role on the change of the coordination compound and the energy of the reactions [8,9]. To predict the shift of equilibrium in the reactions of biologically active molecules, it is necessary to extend the knowledge of solvent influence on these processes. In this context, the aim of this research is to obtain new data of the complexation between β-cyclodextrin (β-CD) and benzoic acid (BA) as a model reaction of the complex formation of hydrophobic molecules with cyclodextrins (CDs) in various media. CDs are cyclic oligosaccharides with a lipophilic internal molecular cavity and a hydrophilic external surface bearing hydroxyl group. Due to their structure, CDs can form water-soluble inclusion complexes with various hydrophobic molecules. The different influence of water and non-aqueous media on molecular complexation suggests the possibility to improve CDs ability to enhance the apparent solubility of hydrophobic molecules by changing the solvent as a medium of occurring processes.

Thus, this research may help developing CD-based pharmaceutical formulations through the modulation of the appropriate solvent mixture that would thus be employed in the industrial field as a means to control the reactions/processes carried out in liquid phases.

The formation of [BA⊂β-CD] molecular complex in solution is described by the following pseudo-chemical equation:

BA_solv_ + β-CD_solv_ ↔ [BA⊂β-CD]_solv_ (1)

The set of new data obtained for the reaction (1) and reported in this paper includes:

- NMR data for the complexation of benzoic acid with β-cyclodextrin in D_2_O-DMSO-*d*_6_ and in D_2_O-EtOH, the stability constants of [BA⊂β-CD] molecular complexes calculated from NMR results;
- Changes in the Gibbs energy of benzoic acid resolvation at the transfer from water to water–dimethylsulfoxide solvents obtained by a method of interfacial distribution of the substance between two immiscible phases;
- Quantum chemical calculations of the interaction energy between β-CD and BA and the structure characteristics of the benzoic acid complex with β-cyclodextrin in vacuum and in water, methanol and DMSO.

The stability of the [BA⊂β-CD] complex in water–ethanol and water–DMSO, obtained by different methods are compared. The thermodynamic parameters of the [BA⊂β-CD] molecular complexation as well as the reagent solvation contributions to the equilibrium of [BA⊂β-CD] complexation in water–ethanol and water–DMSO solvents have been analyzed by using the solvation/thermodynamic approach.

## 2. Results

### 2.1. ^1^H NMR Studies of Inclusion Complex Formation of Benzoic Acid with β-Cyclodextrin in D_2_O, D_2_O-DMSO-d_6_ and D_2_O-EtOH

**Figure 1:**
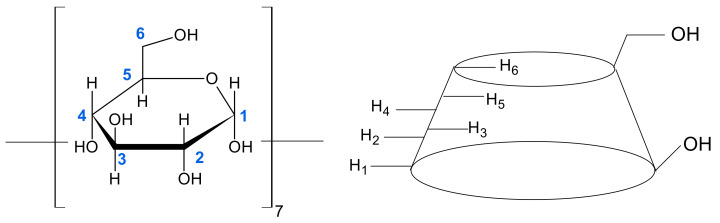
Schematic representation of the β-CD molecule and atom numbering in a single glucopyranose unit.

**Figure 2:**
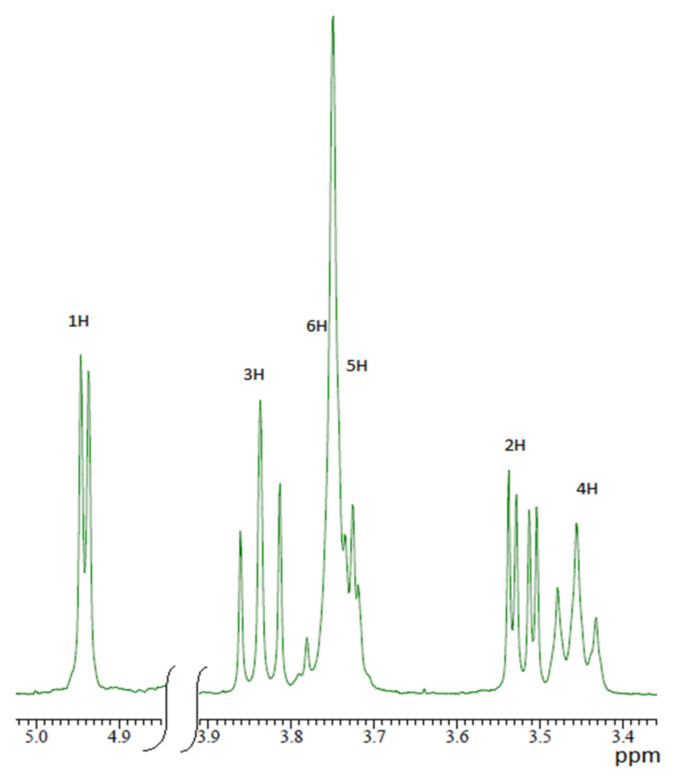
^1^H NMR spectrum of β-CD.

NMR spectroscopy is one of the most important and useful methods to investigate the structure, stability and stoichiometry of host–guest complexes. It provides direct and detailed observation of individual nuclei relevant to the structure and dynamics of the system. The inclusion complexes are formed mainly due to weak interactions (hydrogen bonds, van der Waals forces, etc.), therefore complexation induces relatively small chemical shifts. The most shifted signals in NMR spectrum of complex usually correspond to “interacting” atoms in “host” and “guest” molecules that are close in space. For example, in β-cyclodextrin protons H4 and H2 of the glucopyranose residues are located on the outer side, and protons H3 and H5 on the inner side of the macrocyclic cavity (Figure 1). Therefore, the predominant shifts of H3 and H5 protons (Figure 2) suggest the inclusion of a “guest” molecule into β-CD cavity. By treating the dependencies of observed ^1^H (or ^13^C) chemical shifts from the “host” to “guest” ratio it is possible to obtain the data for stability and stoichiometry of the complexes formed.

**Figure 3:**
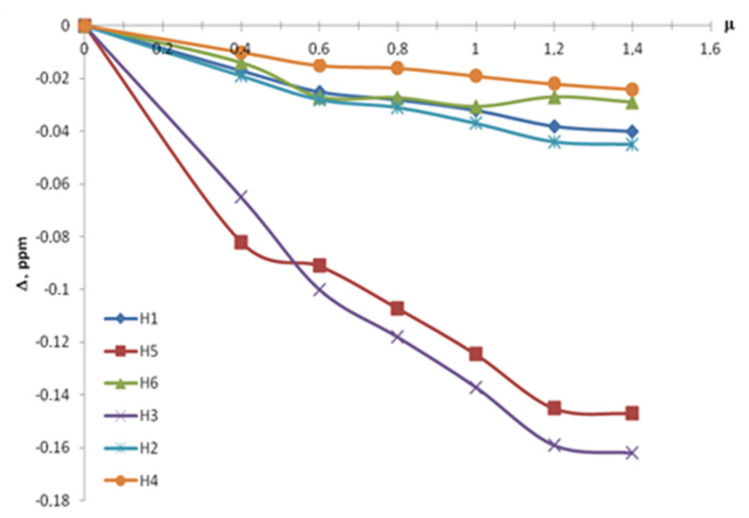
NMR titration curves of the β-CD by BA in D_2_O.

Prior to the studies on the formation of inclusion complexes in mixed solvents, a reference experiment was carried out in D_2_O solution. It consisted of a series of seven points with the different µ = C_BA_/C_β-CD_ ranging from 0 to 1.4. The observed relative chemical shifts of β-CD are given in Table 1 and Figure 3. Labels H1, H2, H3, H4, H5 and H6 refer to the proton position in a glucopyranose unit of β-CD molecule (Figure 1 and Figure 2).

As shown in Figure 3, the inner protons H5 and H3 are the most affected among all the β-CD protons. This confirms the inclusion of a “guest” molecule into the β-CD cavity.

The proximity of the BA aromatic ring to the inner side of β-CD cavity contributes to magnetic anisotropy and shielding effect leading to the upfield shift for H5 and H3 protons of β-CD.

In turn, aromatic protons of BA show perceptible downfield shift, which can be explained by the de-shielding effect of nearby electronegative oxygen atoms arranged inside β-CD cavity. Such a simultaneous chemical shift change could be considered as an evidence of the guest positioning into the cavity of β-CD [10]. Close values of H5 and H3 chemical shifts are usually interpreted as the almost full inclusion of the benzene ring of BA molecule into β-CD cavity, while BA carboxylic group is located outside the cavity [11,12,13]. 

**Figure 4:**
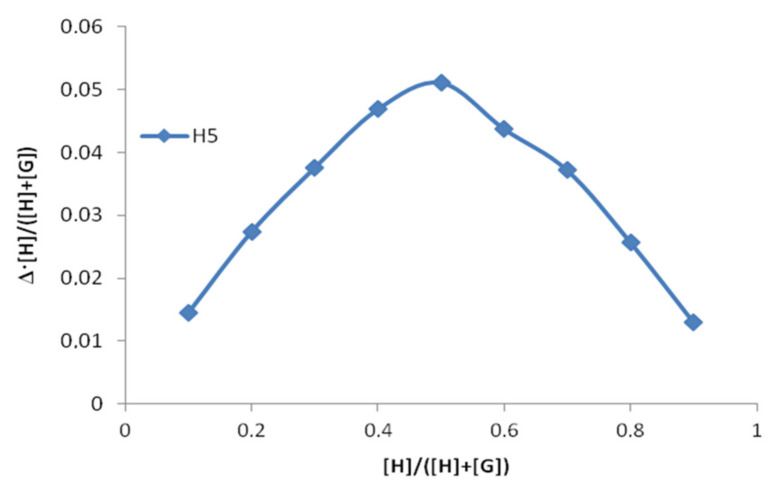
^1^H NMR Job’s plot for the [BA⊂β-CD] system in D_2_O (Shifts refer to β-CD H5 chemical shifts).

These observations are in satisfactory agreement with the results of our calculations. The pattern in the observable chemical shifts changes for the different host to guest ratio assumes the formation of a 1:1 complex. To prove this hypothesis, an additional experiment using Job’s method of continuous variation was carried out. It is the simplest and most intuitive method to prove the stoichiometry when only one type of the complex is formed [14,15,16]. The Job’s plot for the [BA⊂β-CD] system is shown on Figure 4. Maximum at the point [H]/([H] + [G]) = 0.5 confirms the 1:1 complex formation. 

The next part of the work is devoted to the study of the inclusion complex formation between β-cyclodextrin and benzoic acid in D_2_O-DMSO-*d*_6_. 

**Figure 5:**
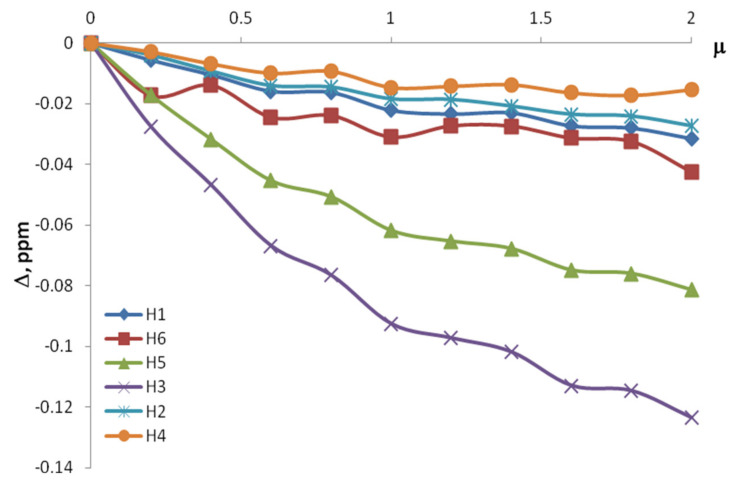
NMR titration curves for BA-β-CD system in D_2_O-DMSO-*d*_6_ (*X*_(DMSO-*d*6)_ = 0.05 mol. fr.).

A preliminary series for *X*_(DMSO-*d*6)_ = 0.24 mol. fr. showed no changes of protons chemical shifts both for β-CD and BA. The maximum change observed for H5 was 0.01 ppm that is within the experimental error. In a series with a molar fraction of 0.15 the statistically significant changes of chemical shifts were observed, but the processing of this data gave unreliable results. Therefore, this series was excluded from further investigation and only series with molar fractions of DMSO-*d*_6_ equal to 0.05, 0.075 and 0.10 were included. The series with *X*_(DMSO-*d*6)_ = 0.075 mol. fr. was carried out in an extended range of BA-β-CD ratio from 0.2 to 2.5. All the raw data on ^1^H chemical shifts for all treated series are given in the Supplementary Materials except for the NMR titration curves for *X*_(DMSO-*d*6)_ = 0.05 mol. fr. presented below (Figure 5).

These data clearly indicate that with an increase of *X*_(DMSO-*d*6)_ in the solvent, the observed chemical shifts tend to decrease (mostly H3 and H5 of β-CD, and to a lesser extent BA protons). We assume that this is due to an increased BA solvation in DMSO-*d*_6_ and competition between solvation and inclusion complex formation processes.

**Figure 6:**
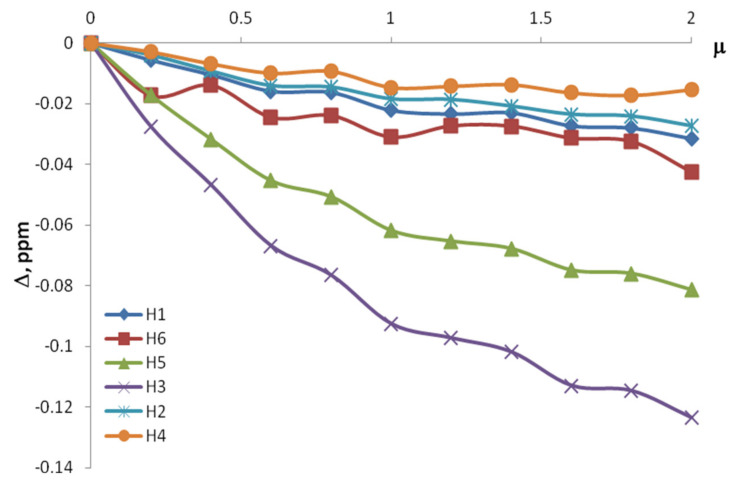
Fitting results for NMR titration curve for BA-β-CD system in D_2_O-DMSO-*d*_6_ (*X*_(DMSO-*d*6)_ = 0.05 mol. fr.).

In order to determine the parameters of inclusion complex formation process (the binding constants and the complexation-induced chemical shifts), the data obtained for all the series (in water and mixed solvents) were fitted by the nonlinear least squares method assuming the one-stage complexation model according to the following equation:

(2) $$\Delta_{obs}=\frac{\Delta_{GH}}{2}\cdot \left(1+\mu +\frac{1}{K\cdot C_{H}}-\sqrt{{\left(1+\mu +\frac{1}{K\cdot C_{H}}\right)}^{2}-4\mu }\right)$$

where Δ*_obs_* is the observable chemical shift, Δ*_GH_* the complexation induced chemical shift of β-CD proton, *C_H_* the concentration of β-CD and *μ* = C_BA_/C_β-CD_. To improve the fitting results, accuracy chemical shift dependencies for both H5 and H3 protons of β-CD were treated simultaneously. Figure 6 shows the fitting results for the treatment of NMR titration curves of the BA β-CD system in D_2_O-DMSO-*d*_6_ (*X*_(DMSO-*d*6)_ = 0.05 mol. fr.). Pearson’s correlation coefficient for H3 and H5 is 0.996 and 0.997, respectively.

Calculation results for D_2_O and D_2_O-DMSO-*d*_6_ are summarized in Table 2. The increase of DMSO molar fraction leads to a monotonic decrease of the induced chemical shifts and binding constants. This may be due to an increase of BA solvation in DMSO-*d*_6_ and the competition between solvation and inclusion complex formation processes. These results are consistent with the assumption made above as well as with the data obtained by other methods.

**Figure 7:**
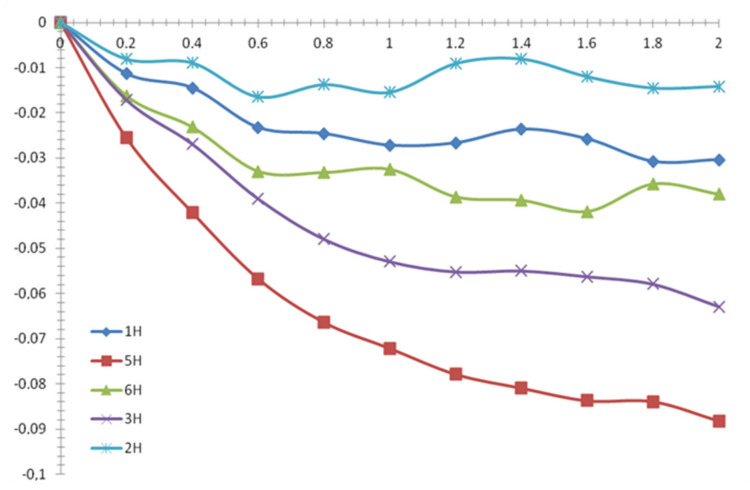
NMR titration curves of the β-CD by BA in D_2_O-EtOH (*X*_(EtOH)_ = 0.075 mol. fr.).

The next three series were investigated in D_2_O-EtOH mixed solvent. Due to the use of non-deuterated ethanol, the EtOH signals in ^1^H NMR spectra were very intensive and, in most cases, partially overlapped with some β-CD signals. A molar fraction of ethanol in a mixed solvent was set to 0.05, 0.075 and 0.10. The further increase of EtOH content prevented accurate measurement of chemical shifts in spectra even when we used various signal suppression techniques, thus *X*_(EtOH)_ = 0.10 mol. fr. was set as a practical upper limit in these series. The observed chemical shifts and NMR titration curves for the BA and β-CD in the case (*X*_(EtOH)_ = 0.075 mol. fr.) are shown in Table 3 and Figure 7. Table 3 shows the data for all β-CD and BA protons. The H3 and H5 protons of β-CD have the highest chemical shifts changes, which is consistent with previous series and confirms the inclusion of the “guest” into β-CD cavity. Benzoic acid protons 2, 6 and 4 have chemical shifts bigger than protons 3 and 5 that are close to the carboxylic group of BA. The most likely it would be explained if only “bottom” part of benzene ring is immersed in β-CD cavity while the carboxylic group of BA protrudes from cavity.

The data obtained were used in calculations of the binding constants and complexation-induced chemical shifts for each D_2_O-EtOH molar ratio using the same procedure as for D_2_O and D_2_O-DMSO-*d*_6_ series. Calculations were carried out for a 1:1 complexation model while simultaneously treating two protons H3 and H5. A two-stage complexation model with one BA and two β-CD molecules was also tested, but it was proven to be erroneous. The results are displayed in Table 4. The increase of the molar fractions of ethanol in the solvent mixture leads to decrease of the binding constant as observed for the D_2_O-DMSO-*d*_6_ series.

Thus, we can conclude that both DMSO-*d*_6_ and EtOH in mixed solvent compete with BA for the β-CD cavity. On the other hand, despite these components decrease the values of binding constants in solution, we found they could favor the formation of inclusion complexes as they significantly increase the yields when we prepared the complexes in the solid state.

### 2.2. Quantum Calculation Results 

#### 2.2.1. Geometry Optimization and Properties of β-CD and BA 

**Figure 8:**
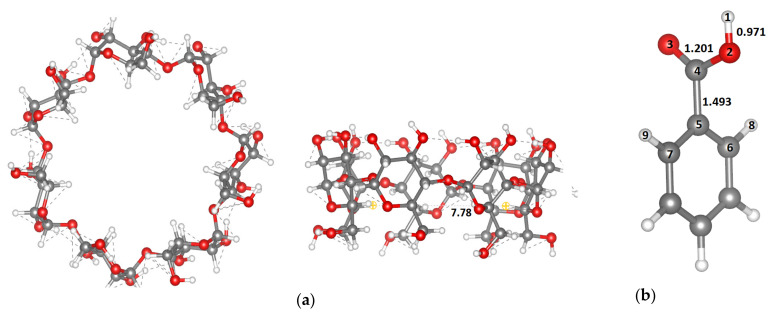
Optimized structures of: β-CD (**a**) and BA (**b**) calculated by GFN2-xTB method. Colors: grey—C, red—O, white—H; all key distances are in Å.

The optimized structures of β-CD and BA obtained with the GFN2-xTB method are presented in Figure 8. As for the β-CD structure, the outer diameters range from 13.33 to 14.36 Å, while the inner diameter was found to be 7.78 Å, which is in good agreement with the literature [17].

#### 2.2.2. [BA⊂β-CD] Complex Formation

**Figure 9:**
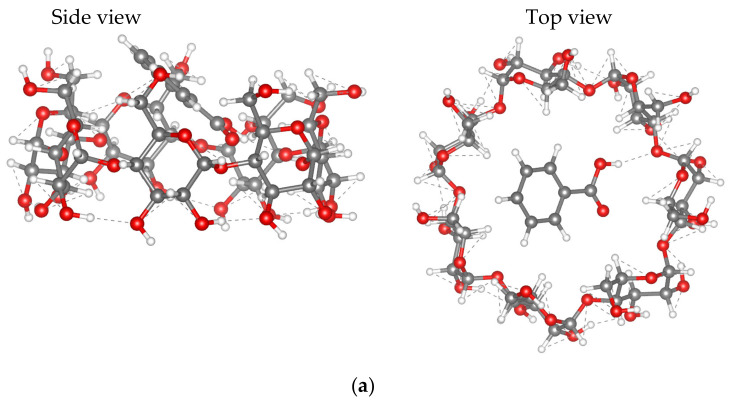

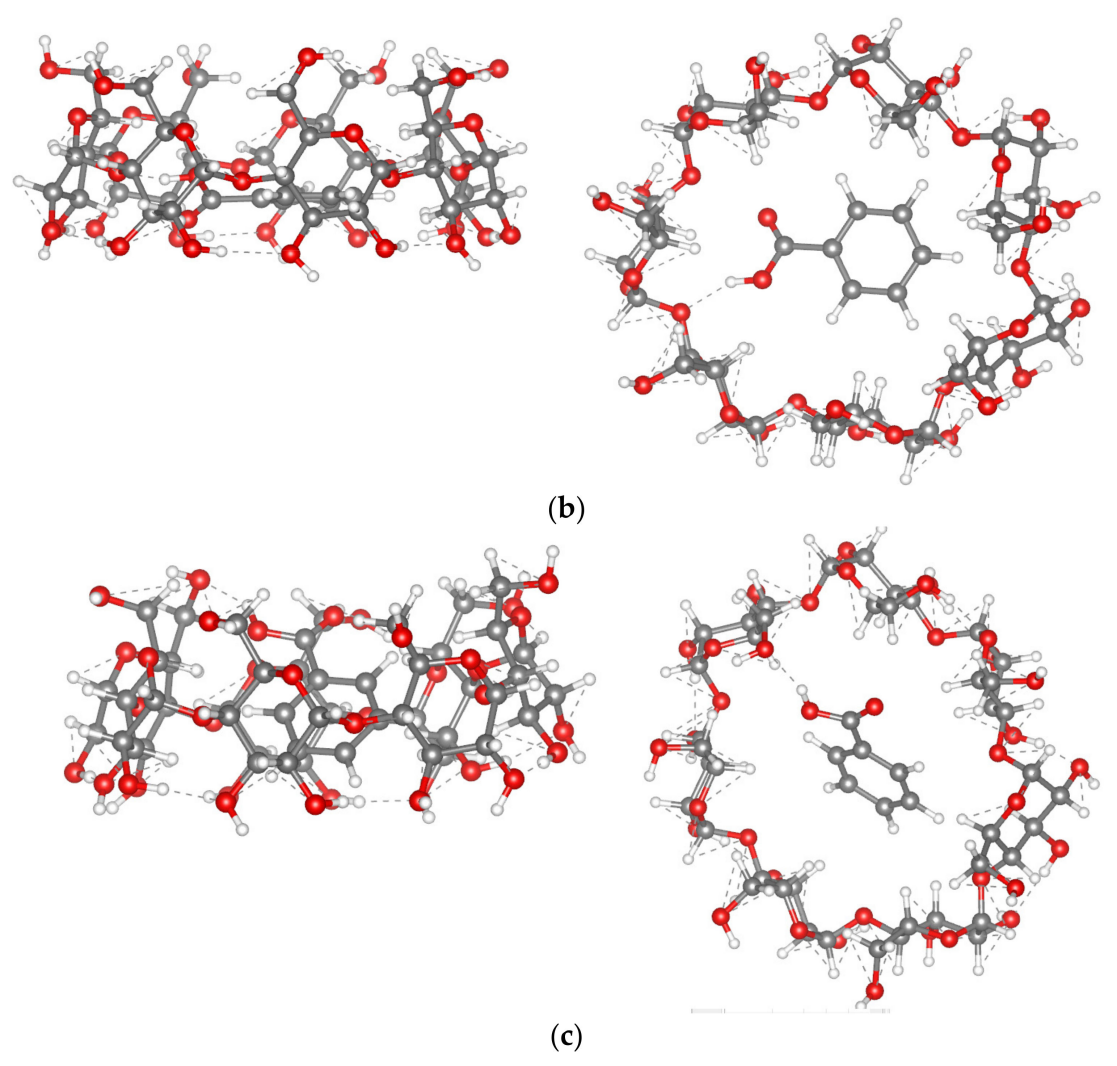
Three [BA⊂β-CD] interaction configurations: (**a**) HF, (**b**) PR and (**c**) TF.

From iMTD-GC sampling results, we optimized and calculated the interaction energy for three [BA⊂β-CD] configurations: (i) the “head first” configuration (denoted as HF), in which the BA molecule is vertically located with the –COOH group pointed towards the CD cavity (Figure 9a); (ii) the “parallel” configuration (denoted as PR) in which the BA molecule is parallelly located to the CD (Figure 9b); and (iii) the “tail first” configuration (denoted as TF), corresponding to the vertical position of BA with the phenyl group pointed towards the CD cavity (Figure 9c). All xyz coordinate files of the structures shown in Figure 8 and Figure 9 can be found in the Supplementary Materials. Comparison and evaluation of interaction energy will reveal which interaction configuration is favored. Table 5 shows the calculated interaction energy for the HF, PR and TF configurations in vacuum and in different solvents (in water, methanol and DMSO). Methanol has been selected as the simplest representative of the homologous series of monohydric alcohols.

The results show that the formation of the TF configuration corresponds to the most negative interaction energy value in the vacuum, as well as in all solvents. In other words, in all cases, the TF is the most preferred configuration. The increase in E_int_ in solvents compared with that in vacuum likely results the solvation effect, which reduces the interaction between β-CD and BA. 

Analysis of the influence of the solvent on the interaction between β-CD and BA shows that the E_int_ decreases in the order: vacuum > water > methanol > DMSO. This indicates that β-CD and BA interact most strongly in vacuum. The presence of a solvent considerably reduces the interaction between β-CD and BA. Furthermore, among the investigated solvents, water is the most favorable for the complexation, whereas DMSO is much less preferable. This finding allows us to predict that in a binary solvent consisting of water and an organic solvent such as methanol or DMSO, as the organic solvent content increases the stability of the complex will decrease.

The E_int_ values for all examined interaction configurations in vacuum and solvents are not excessively negative, ranging from −2.54 to −102.86 kJ mol^−1^. As a result, the interaction between β-CD and BA can be considered as a physical interaction, which is mainly governed by non-covalent interactions such as dispersion interaction and hydrogen bonding (as shown by the dot lines in Figure 9). It should be emphasized that, in the GFN2-xTB method, the parameters were specifically optimized for non-covalent interactions. 

To get further insight into the nature of the host-guest interaction, significant changes in geometrical parameters and electronic properties of the BA molecule before and after the interaction with CD have been analyzed. The results are shown in Table 6. 

Compared to the non-complexed molecule, the structure of BA slightly changes when placed in the cavity of β-CD. The most significant difference in geometrical structure of BA before and after interacting with β-CD is obtained for H1O2C4C5 and O3C4C5C6 dihedral angles and the H1-OH bond length. The COOH group of BA slightly turns away from the plane of the benzene ring while the H1-OH bond is elongated. As for TF configuration in vacuum, the H1-O (in the -CH_2_OH group of β-CD) bond length is 1.847 Å, which is quite close to the hydrogen bond lengths in the BA-BA dimer structure (1.66–1.83 Å) [18]. In this case, the charge transfer from β-CD to the BA molecule is remarkable compared to that in the presence of solvents. This is ascribable to the formation of the hydrogen bonds between β-CD and BA in the vacuum. However, in the presence of solvents, the hydrogen bonds between β-CD and BA are not clearly observed. This finding is in good agreement with the calculated interaction energy for the TF configurations in solvents. The E_int_ for the TF configuration was determined to be only −19.09 kJ mol^−1^ in the DMSO solvent, which is much higher than the E_int_ in the vacuum (−102.86 kJ mol^−1^).

### 2.3. Interfacial Distribution-Study of Reagents Solvation State 

The calculation of the distribution coefficients of benzoic acid between immiscible phases such as water–dimethylsulfoxide and n-hexane, and the change in the Gibbs energy during its transfer from water to water–dimethylsulfoxide solvents were carried out according to the equations:

K_1_ = [BA]^Hex^/[BA]^H2O^ (3)

K_2_ = [BA]^Hex^/[BA]^H2O-DMSO^ (4)

∆*_tr_G*^0^(BA)^H2O^^-DMSO^ = RTln([BA]^H2O^/[BA]^H2O-DMSO^) = RTlnK_2_/K_1_ (5)

The equilibrium concentrations of benzoic acid in aqueous and mixed solutions of [BA]^H2O^ and [BA]^H2O-DMSO^ were calculated by KEV software [19] using the corresponding dissociation constants [20]. In the n-hexane layer ([BA]^Hex^) was calculated as the difference between the total concentration of benzoic acid and the total concentration of benzoic acid in the aqueous–organic layer after mixing with hexane, assuming that the volume of the reaction medium remained constant during the experiment.

The errors are reported as the standard deviation for 3–5 parallel experiments. The values of the Gibbs energy of benzoic acid change (∆*_tr_G*^0^(BA)) were assumed to be standard values, due to the low concentration conditions and the absence of concentration dependences of the distribution coefficients of benzoic acid in the experimental conditions.

The concentration conditions of the experiments, the distribution coefficients of BA in the Hex-H_2_O and Hex-H_2_O-DMSO systems and the Gibbs energy change of benzoic acid are shown in Table 7. The ∆*_tr_G*^0^(BA) values have been used in the analysis of the reagent solvation contributions into the Gibbs energy changes of the [BA⊂β-CD] molecular complex formation (Section 3.3).

## 3. Discussion

### 3.1. Thermodynamic Parameters of the [BA⊂β-CD] Complexes Formation in H_2_O-DMSO and H_2_O-EtOH Solvents

The thermodynamic characteristics of the formation of complexes of inclusion of cyclodextrins with BA and its derivatives in water and the structure of the resulting complexes in the crystalline state are presented in numerous literature sources [10,11,12,21,22,23,24,25,26,27,28,29,30,31,32,33,34,35,36,37,38,39,40,41,42,43]. Most of them indicate the formation of complexes with a 1:1 stoichiometric ratio due to the predominance of van der Waals interactions and H-binding between guest molecules and CDs. The complexation of native and hydroxypropylated α-, β- and γ-cyclodextrins with benzoic acid and its derivatives has been studied in aqueous solution by isothermal calorimetry titration, densimetry, ^1^H NMR and UV spectroscopy at 298.15 K [10,21,22,23,24,25,26]. In addition, NOE, ^1^H NMR and ^13^C NMR were used to study the structure of cyclodextrin inclusion complexes with benzoic acid and its derivatives [11,30,40]. 

Computational chemistry methods were used to study the structure and stability of inclusion complexes involving cyclodextrins [39,40,44]. The results of semi-empirical calculations performed in [38,44] using the AM1 method for the inclusion complexes of α-and β-cyclodextrins with benzoic acid and phenol show that the complexes of α-cyclodextrin with both “guest” molecules in the “substituent groups” position are more stable than in the “benzene ring first” position, while the complex of β-cyclodextrin with phenol in the “benzene ring-first” position is more stable 

β-CD inclusion complex with benzoic acid was crystallographically characterized [10]. Two β-CD co-crystallize with two BA molecules, 0.7 ethanol molecules and 20.65 water molecules in the triclinic space group [2(C6H10O5)·7·2(C7H6O2)·0.7 (C2H6O)·20.65H2O]. In the crystal lattice, β-CD forms dimers O-2(m)_1/O-3(m)_1⋯O-2(n)_2/O-3 (n)_2, stabilized by direct hydrogen bonds (intradimer) and indirect hydrogen bonds O-6(m)_1⋯O-6 (n)_2 with one or two water molecules bound together (interdimer).

The thermodynamic parameters (lg*K*^0^, Δ*_r_H*^0^, Δ*_r_G*^0^, *T*Δ*_r_S*^0^) of the complexation of BA with β-CD in the H_2_O-DMSO and H_2_O-EtOH mixtures are displayed in Table 8. The values of the stability constants of the inclusion complex [BA⊂β-CD] in water obtained by different methods are in satisfactory agreement with each other and with the literature data reported for similar experimental conditions.

Thermodynamic parameters of the [BA⊂β-CD] complexes formation in [45] were calculated by the HEAT program [46]. Mathematical processing of experimental data by the HEAT was also successfully used in the study of the formation of low stability molecular and ionic complexes formed by macrocyclic 18-crown-6 ether in water–organic solvents [47,48].

The ranges of the molar fractions of DMSO and EtOH in water–organic solvents were limited due to the lowest complex stability in mixed solvents than in water and hence making it impossible to calculate reliable thermodynamic parameters for [BA⊂β-CD] molecular complex in mixed solvents with high concentration of DMSO or EtOH. Despite these limitations, the obtained results allowed to analyze the effect of the cosolvent on the change in the thermodynamic parameters of the reaction (1).

It was found that from 0.00 to 0.05 mol. fr. Of DMSO the stability of the [BA⊂β-CD] complex slightly decreases, and a further increase in the DMSO content to 0.10 mol. fr. leads to a change in the values of lg*K*^0^ within the experimental error (Table 8). We also observe that the formation of the [BA⊂β-CD] complex becomes slightly less exothermic.

**Table 8:** Thermodynamic parameters for the reaction of formation of [BA⊂β-CD] complexes (lg*K*^0^, Δ*_r_H*^0^, Δ*_r_G*^0^ and *T*Δ*_r_S*^0^) in H_2_O-DMSO and H_2_O-EtOH solvents of variable composition at *T* = 298.15 K.

*X*, mol. fr.	lg*K*^0^	–Δ*_r_H*^0^ kJ mol^–1^	–Δ*_r_G*^0^ kJ mol^–1^	–TΔ*_r_S*^0^ kJ mol^–1^	Experimental Method	Reference
0.00	2.4 ± 0.10 ^(2)^	12.2 ± 0.5	13.7 ± 0.6	−1.5 ± 0.6	Isothermal titration calorimetry (pH 3.6)	[45]
	1.85 ± 0.16 ^(3)^	-	10.6 ± 0.8	-	UV–Vis spectroscopy (pH 3.6)	[45]
	1.94 ^(1)^	-	11.3	-	Solubility method (pH 2.9)	[37]
	2.60 ± 0.10 ^(2)^	13.4 ± 0.4	14.7 ± 0.7	−1.3 ± 0.8	Isothermal titration calorimetry (acidic medium)	[21]
	2.50 ^(5)^	22.3 ± 0.8	14.4	7.9	Circular dichroism spectroscopy	[49]
	2.50 ^(4)^	-	14.2	-	1H NMR (acidic medium)	[12]
	2.59 ^(4)^	-	14.8	-	1H NMR	This work
	2.40 ± 0.2 ^(6)^	-	13.5 ± 1.1	-	Densitometry (acidic medium)	[12]
	2.10 ± 0.4 ^(2)^	32 ± 2.7	12 ± 0.5	20	Isothermal titration calorimetry	[36]
**H_2_O-DMSO**						
0.05	1.67 ± 0.2 ^(2)^	16.8 ± 0.3 *	9.5 ± 0.36	7.3 ± 0.47 *	Isothermal titration calorimetry (pH 3.6)	[45]
	1.78 ± 0.2 ^(3)^	-	10.2 ± 0.2	-	UV–Vis spectroscopy (pH 3.6)	[45]
	1.94 ± 0.45 ^(2)^	16.9 ± 1.4	11.07	5.2	Isothermal titration calorimetry (pH 1.65)	[45]
	2.57 ^(4)^	-	14.7	-	1H NMR	This work
0.075	2.2 ^(4)^	-	-	-	^1^H NMR	This work
0.10	1.86 ± 0.41 ^(2)^	10.9 ± 0.9 *	10.9 ± 0.9	0.3 ± 1.3 *	Isothermal titration calorimetry (pH 3.6)	[45]
	1.86 ± 0.41 ^(3)^	-	10.9 ± 0.9	-	UV–Vis spectroscopy (pH 3.6)	[45]
	1.85 ± 0.32 ^(2)^	10.1 ± 0.2	10.6 ± 0.9	–0.5	Isothermal titration calorimetry (pH 1.65)	[45]
	1.49 ^(4)^	-	8.5	-	1H NMR	This work
**H_2_O-EtOH**						
0.05	2.47 ^(4)^	-	-	-	^1^H NMR	This work
0.075	2.40 ^(4)^	-	-	-	^1^H NMR	This work
0.10	1.90 ± 0.10 ^(2)^	36.8 ± 0.2	10.8 ± 0.6	26.0 ± 0.7	Isothermal titration calorimetry (pH 3.6)	[50]
	2.29 ^(4)^	-	-	-	1H NMR	This work
0.20	0.70 ± 0.10 ^(2)^	44.3 ± 0.6	3.9 ± 0.6	40.4 ± 0.8	Isothermal titration calorimetry (pH 3.6)	[50]

^(1)^ Phase solubility method; ^(2)^ Isothermal calorimetry of titration; ^(3)^ UV spectroscopy; ^(4) 1^H NMR spectroscopy; ^(5)^ Circular dichroism spectroscopy; ^(6)^ Densitometry; * calculated using the HEAT program taking into account the lg*K*^0^ values obtained from the UV-Vis spectroscopy.

An increase in the EtOH content in the H_2_O-EtOH solvent to 0.20 mol. fr. leads to a decrease in the stability of the [BA⊂β-CD] complex. At the same time, the exothermicity of complexation increases, which is accompanied by an increase in the numerical values of the entropy component of the change in the Gibbs energy of complexation. With a molar fraction of ethanol *X*_(EtOH)_ = 0.20 mol. fr. the enthalpy and entropy contributions to the change of Gibbs energy compensate each other, which leads to a decrease in the stability of the complex to lg*K*^0^ = 0.70 ± 0.10.

The analysis of the effect of the compositions of the H_2_O-EtOH and H_2_O-DMSO solvents on the stability of the molecular complex [BA⊂β-CD] shows that the stability of the complex in the H_2_O-DMSO varies within the experimental error, while decreases with increasing of ethanol content in H_2_O-EtOH solvents.

**Figure 10:**
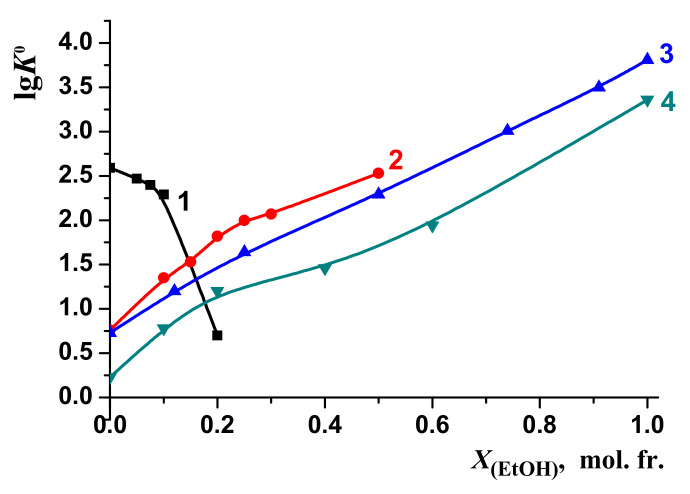
Effect of H_2_O-EtOH solvents on the stability of molecular complexes: 1—[BA⊂β-CD]; 2—[3Gly18C6], [51,52]; 3—[Gly18C6], [53]; 4—[Phe18C6], [56].

**Figure 11:**
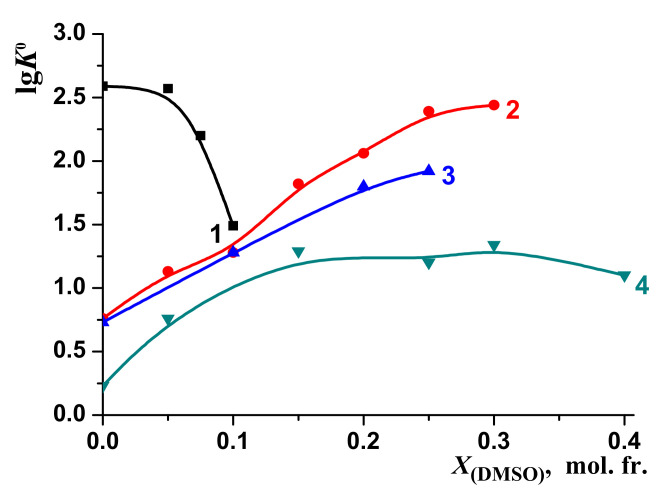
Effect of H_2_O-DMSO solvents on the stability of molecular complexes: 1—[BA⊂β-CD]; 2—[3Gly18C6], [52]; 3—[Gly18C6], [54]; 4—[Phe18C6], [57].

The changes in the stability of the molecular complexes [BA⊂β-CD] were compared with those of the molecular complexes of 18-crown-6 ether (18C6) with glycyl–glycyl–glycine (3Gly), [3Gly18C6] [51,52], glycine (Gly), [Gly18C6] [53,54] and phenylalanine (Phe) [Phe18C6] [55,56] during the transfer from water to water–organic solvents (Figure 10 and Figure 11). These objects were selected for comparison of the “guest–host” molecular complexes formation in aqueous-organic solvents. However, we observed a different effect of solvents on these processes: the increase of EtOH and DMSO concertation leads to increasing of the stability of the molecular complexes [3Gly18C6], [Gly18C6] and [Phe18C6]. On the contrary, the stability of [BA⊂β-CD] decreases.

Previous studies [51,52,56,57,58,59] showed that the stability of complexes tends to increase as the content of non-aqueous components of solvents increases in line: lg*K*^0^[Phe18C6] < lg*K*^0^[Gly18C6] < lg*K*^0^[3Gly18C6].

**Figure 12:**
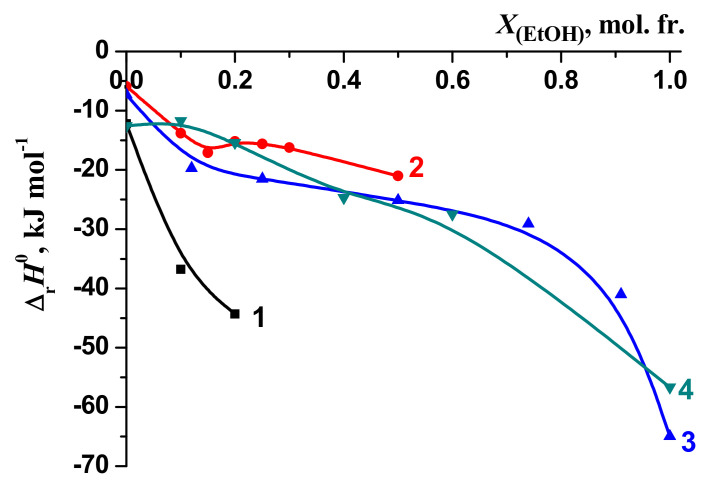
Change in the enthalpy of complex formation reactions in H_2_O-EtOH solvents: 1—[BA⊂β-CD]; 2—[3Gly18C6], [51,52]; 3—[Gly18C6], [53]; 4—[Phe18C6], [56].

**Figure 13:**
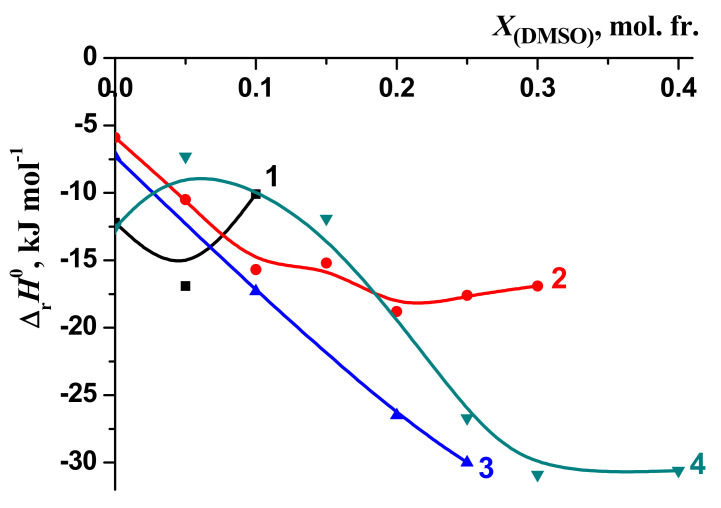
Changes in the enthalpy of complex formation reactions in H_2_O-DMSO solvents: 1—[BA⊂β-CD];.2—[3Gly18C6], [54], 3—[Gly18C6], [58]; 4—[Phe18C6], [57].

The changes in the enthalpy of the reactions of the formation of complexes [BA⊂β-CD], [3Gly18C6] [51,52], [Gly18C6] [53,54,55] and [Phe18C6] [56,57] when transferred from water to water–organic solvents are shown in Figure 12 and Figure 13. In the case of water–ethanol solvent the increase of the exothermicity of the reaction is observed for all examined molecular complexes [BA⊂β-CD], [3Gly18C6], [Gly18C6] and [Phe18C6].

The highest increase in the exothermicity of the reactions (Figure 12 and Figure 13) has been observed when passing from water to ethanol, whilst the smaller value is obtained in DMSO. 

The influence of H_2_O-EtOH and H_2_O-DMSO solvents on molecular complex formation is explained on the base of solvation-thermodynamic approach and is described below.

### 3.2. Solvation/Thermodynamic Approach 

The nature, composition and structure of the solvent are the fundamental parameters that determine the stability of a complex, its composition, the rate of complex formation, as well as the mechanism of the complexation reaction.

A wide body of experimental evidence has been accumulated, showing the extremely important role of the solvent as a means of controlling the chemical process [2,3,60]. This has spurred the development of theoretical and experimental studies aimed at studying the functional behavior of the solvent in chemical reactions and establishing general patterns of the solvent influence on the thermodynamics of complex formation.

The influence of the solvent on the shift of the chemical equilibrium is so diverse that a general theory describing the role of the solvent in chemical processes has not yet been developed, despite a large amount of research. Here, we will focus on the role played by the solvent without aspiring to be exhaustive owing to the abundance of literature data [61,62,63,64,65,66,67,68,69,70]. 

From this point of view, solvation approaches to the explanation of the causes of changes in the thermodynamic characteristics of complexation reactions with varying solvent composition are of universal importance. 

A universal approach based on the thermodynamic characterization of the solvation of all reagents is more promising for creating a scientific basis for the use of a solvent as a means of controlling liquid-phase processes. At the same time, the thermodynamic parameters (Δ*G*^0^, Δ*H*^0^, Δ*S*^0^) of the reaction and solvation (or transfer) of the complexing ion, ligand, and coordination compound are determined; their contributions to the total solvent effect have been analyzed to determine which reagent solvation is crucial.

In accordance with the thermodynamic cycle the solvate–thermodynamic effect of the reaction Δ*_tr_Y*^0^*_r_* = Δ*_r_Y*^0^*_S_* − Δ*_r_Y*^0^*_W_*, i.e., the change in the thermodynamic characteristic of the reaction in a non-aqueous solvent (compared to an aqueous one) is the result of three solvation contributions:

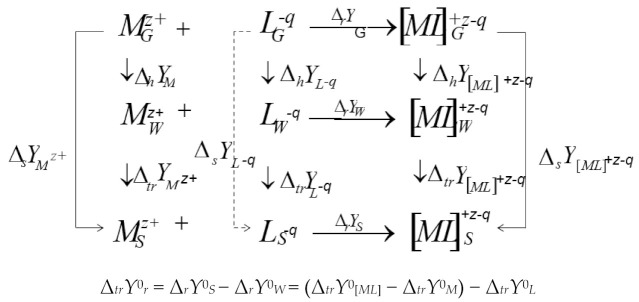
(6)
where: Δ*Y*^0^ = Δ*G*^0^, Δ*H*^0^, Δ*S*^0^.

This relationship is the basis of the solvation approach to the description of the role of the solvent in complexation reactions, which we will use in the following paragraphs when analyzing the literature data on the thermodynamics of complexation reactions in binary mixtures of water–organic and non-aqueous solvents.

### 3.3. The Thermodynamic Characteristics of the Molecular Complexation and Solvation of the Reagents: A Solvation-Thermodynamic Analysis

**Figure 14:**
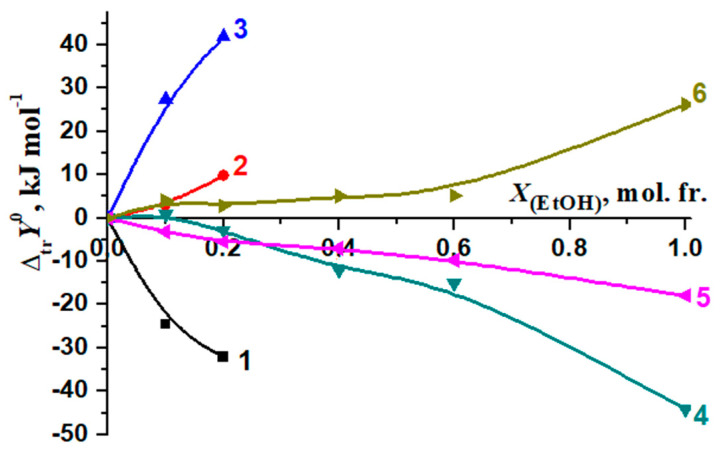
Changes in the thermodynamic parameters of complexation reactions during transfer from water into H_2_O-EtOH solvents: [BA⊂β-CD], 1—Δ*_tr_H*^0^, 2—Δ*_tr_G*^0^, 3—−*T*Δ*_tr_S*^0^; [Phe18C6], 4—Δ*_tr_H*^0^, 5—Δ*_tr_G*^0^, 6—−*T*Δ*_tr_S*^0^ [56].

**Figure 15:**
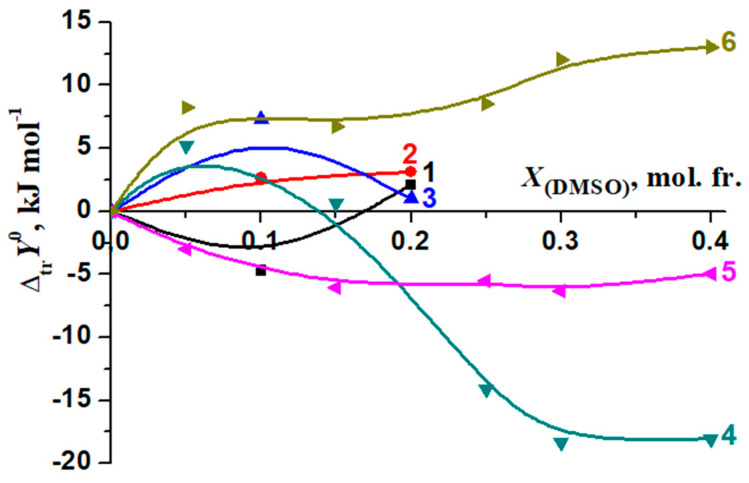
Changes in the thermodynamic parameters of complexation reactions during transfer from water into H_2_O-DMSO: [BA⊂β-CD], 1—Δ*_tr_H^0^*, 2—Δ*_tr_G^0^*, 3—−*T*Δ*_tr_S^0^*; [Phe18C6], 4—Δ*_tr_H^0^*; 5—Δ*_tr_G^0^*; 6—−*T*Δ*_tr_S*^0^ [57].

Changes in the thermodynamic parameters of the reactions of formation of molecular complexes [BA⊂β-CD] and [Phe18C6] [56,57] during transfer from water to water–organic solvents are shown in Figure 14 and Figure 15.

In H_2_O–EtOH, there is a significant difference in the change in the corresponding thermodynamic transfer functions of complexation [BA⊂β-CD] and [Phe18C6] (Figure 14). On the contrary, H_2_O–DMSO solutions with a 0.00, 0.10 mol. fr. of DMSO did not show a significant differentiating effect on the change in the thermodynamic parameters of the reactions of the formation of [BA⊂β-CD] and [Phe18C6] (Figure 15).

There are some differences in complexation between BA and β-CD in water and in a water–organic solvent: the part of the inner cavity of β-CD in which the BA molecule is located may be more hydrophobic in water than in the water–organic mixture, and this may be the reason for the increase in the complexation exothermicity when passing from water to water–ethanol mixtures. Analysis of the solvation contributions of reagents to the change in the Gibbs energy and enthalpy of the molecular complex formation reaction can shed light on this phenomenon.

**Figure 16:**
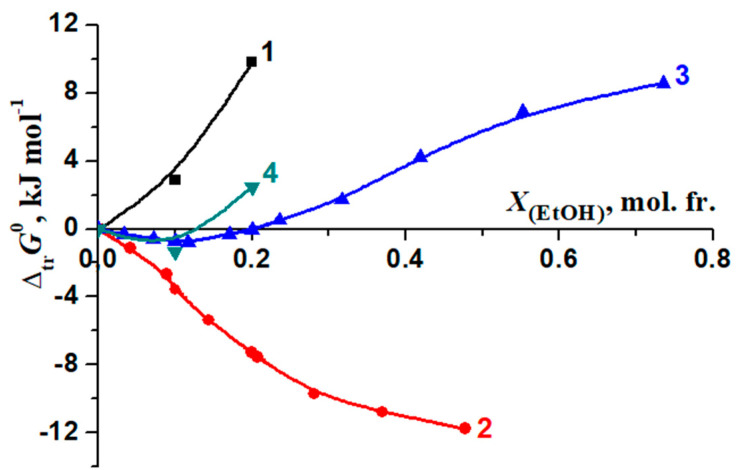
Influence of water–ethanol solvents on the Gibbs energy change of the reaction of formation of [BA⊂β-CD] and solvation of reagents during the transition from H_2_O to H_2_O-EtOH: 1—Δ*_tr_G_r_*^0^; 2—Δ*_tr_G*^0^ (BA) [71]; 3—Δ*_tr_G*^0^ (β-CD) [72]; 4—Δ*_tr_G*^0^ [BA⊂β-CD].

**Figure 17:**
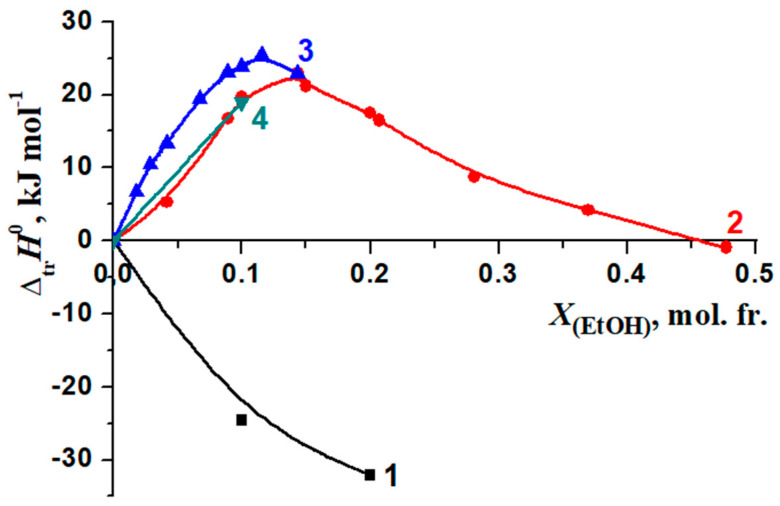
The effect of the water–ethanol mixture on the enthalpy of the reaction change of the formation of [BA⊂β-CD] and the solvation of reagents during the transition from H_2_O to H_2_O-EtOH solvents: 1—Δ*_tr_H_r_*^0^; 2—Δ*_tr_H*^0^ (BA) [71]; 3—Δ*_tr_H*^0^ (β-CD) [73]; 4—Δ*_tr_H*^0^ [BA⊂β-CD].

The relations of the thermodynamic characteristics of the reaction of the formation of [BA⊂β-CD] and the solvation of the reagents are shown in Figure 16 and Figure 17.

The Δ*_tr_G*^0^ (β-CD) and Δ*_tr_G*^0^ [BA⊂β-CD] values are close to zero at concentrations *X*_(EtOH)_ = 0.00 ÷ 0.15 mol. fr., indicating that the decrease in the stability of the complex is determined by an increase in the solvation of BA (Δ*_tr_G*^0^(BA)) (Figure 16).

**Figure 18:**
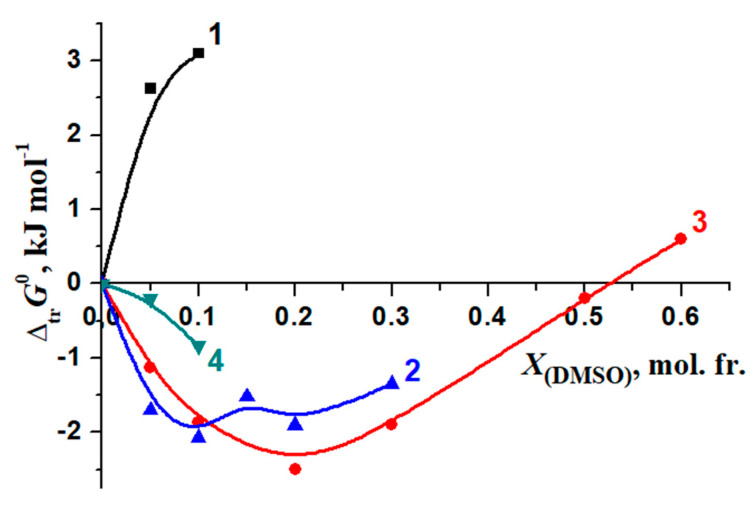
Effect of a water–dimethylsulfoxide mixture on the Gibbs energy changes of the benzoic acid complexation with β-cyclodextrin and the solvation of reagents in H_2_O-DMSO: 1—Δ*_tr_G_r_*^0^; 2—Δ*_tr_G*^0^ (BA); 3—Δ*_tr_G*^0^ (β-CD) [74]; 4—Δ*_tr_G*^0^ [BA⊂β-CD].

When a small amount of EtOH is added to the solvent (*X*_(EtOH)_ < 0.12 mol. fr.), the increase in the exothermicity of the complex formation reaction (Δ*_tr_H_r_*^0^) is determined by the de-solvation of both β-cyclodextrin and BA (Figure 17). The ratios of reagents solvation contributions into the Gibbs energy change of the complexation in water–DMSO are slightly different from that in H_2_O-EtOH (Figure 18).

Solvation increases almost equally for both reagents (BA and β-CD) in the initial stage of DMSO addition to water. As a consequence, a decrease of [BA⊂β-CD] complex stability has been observed, but the increase of the solvation of the molecular complex leads to the decrease of this effect. 

## 4. Materials and Methods

### 4.1. Materials

The following chemical reagents were used in this work:

- Benzoic acid (C_6_H_5_COOH) (“chemically pure” grade), used without additional purification. The purity of reagents was declared by the manufacturer >99% by weight.
- β-cyclodextrin (C_42_H_70_O_35_) from Sigma-Aldrich Saint (Louis, MO, USA) with a CD content of ≥99% was used without additional purification;
- Dimethylsulfoxide (C_2_H_6_OS) was purified by distillation according to the method [75] before use. The DMSO content was 99.4 wt.%. The residual water content in the organic solvents used was taken into account when preparing the solutions.
- Deuterated water and dimethylsulphoxide (atomic fraction of deuterium more than 99.9%) were purchased from Sigma-Aldrich USA. Ethyl alcohol (C_2_H_5_OH) (96% by vol.) was purified by distillation at the atmospheric pressure.

The solutions were prepared by the weight method according to exact weights. The analytical scales of the brand AUW220D (SHIMADZU) were used. For the preparation of solutions, fresh bidistillate water was used.

### 4.2. Methods

#### 4.2.1. NMR Spectroscopy

All NMR ^1^H spectra were recorded on JNM-ECA-400 (400 MHz, «JEOL», Tokyo, Japan) NMR spectrometer at the temperature 298 K using DSS (Sodium trimethylsilylpropanesulfonate) as the external standard.

When studying the inclusion complex formation, three types of series were run. One series was prepared in pure deuterated water. Four series were done in a D_2_O-DMSO-*d*_6_ mixed solvent (with DMSO-*d*_6_ molar fractions 0.05, 0.075, 0.10 and 0.15) and three series in a D_2_O-EtOH mixed solvent (with molar fractions of EtOH 0.05, 0.075, and 0.10). In each series, the β-CD concentration held constant (0.0135 mol/L), while the benzoic acid concentration was variable.

#### 4.2.2. Models and Computational Methods

All geometry and energy calculations have been performed by using an accurate and broadly parametrized self-consistent tight-binding quantum chemical method with multipole electrostatics contribution called GFN2-xTB method (short for “Geometry, Frequency, Noncovalent, eXtended tight-binding”). The GFN2-xTB method includes hydrogen bonding, halogen bonding as well as dispersion corrections (via D4 London dispersion model). This method has been parameterized for all spd-block elements and the lanthanides up to Z = 86 [76,77,78]. In this work, geometry optimization was performed with an electronic temperature of 300K, integral cutoff of 0.25 × 10^2^, SCF convergence of 0.1 × 10^−5^ Ha and wavefunction convergence of 0.1 × 10^−3^ e. 

When BA interacts with β-CD, there are numerous binding positions. The interaction between β-CD and BA has been studied by the following scheme: First, searching of the most preferable configurations was performed using meta-dynamics (MTD) simulations with an additional genetic z-matrix crossing (GC) approach for the generation of conformer/rotamer ensembles [79]. This method combines forcefield speed with almost quantum mechanical accuracy [80]. Next, the most favorable (the best) configurations generated from iMTD-GC algorithm were fully optimized by GFN2-xTB method. The results obtained by GFN2-xTB method reach an accuracy remarkably close to the DFT reference, justifying their application for efficient binding site screening [81].

The nature of the interaction between β-CD and BA was estimated based on interaction energy (E_int_) which is calculated as follows:

E_int_ = E[BA⊂β-CD] − E(β-CD) − E(BA) (7)

where E[BA⊂β-CD], E(β-CD) and E(BA) are the energy of the [BA⊂β-CD] configuration, β-CD and BA, respectively.

The interaction energy can be considered as a critical thermodynamic criterion to estimate the ability and the extent of the process. In addition, a significant change in the geometrical structures of BA was also analyzed. The population analysis, including charge transfer and bond orders were reported and discussed for a detailed description of the interaction process. Furthermore, the influence of the different solvents was also included via the analytical linearized Poisson–Boltzmann (ALPB) model.

#### 4.2.3. Interfacial Distribution-Study of Reagents Solvation State

The distribution coefficients of benzoic acid in water and its mixtures with DMSO are determined by the method of interfacial distribution of the substance between two immiscible phases: an aqueous or water–dimethylsulfoxide solution and *n*-hexane. The applicability of this method for similar studies, as well as experimental confirmation of the immiscibility of a water–dimethylsulfoxide solvent (*X*_(DMSO)_ = 0.0–0.5 mol. fr.) with *n*-hexane are given in [82,83].

During the experiment, equal aliquots of an aqueous organic solution of benzoic acid and n-hexane were placed in a flask with polished lids. The contents of the flask were stirred with a magnetic stirrer for 8 hours at a constant temperature (298.2 ± 0.1 K). After settling (15 h), a sample of the lower layer of the heterogeneous system (benzoic acid + solvent H_2_O–DMSO) was taken, in which the equilibrium concentration of benzoic acid in the water–dimethylsulfoxide layer ([BA]^H2O-DMSO^) was spectrophotometrically determined.

All measurements were carried out on a two-beam UV spectrophotometer SPECORD M400 (Shimadzu), at a wavelength of 273.0 nm in cuvettes with a thickness of 10 mm at a concentration of benzoic acid from 1.133 × 10^−4^ to 6.276 × 10^−4^ mol/L in the optical density range 0.15–0.6.

## 5. Conclusions

The experimental results are in accordance with the theoretical calculations performed for the inclusion studies of BA in β-CD and complement each other. Stability constants obtained by NMR agree with those calculated from calorimetric measurements. In turn, the calorimetric and NMR data for [BA⊂β-CD] stability also confirm that the addition of DMSO (up to 0.10 mol. fr.) leads to the most significant decrease in the stability of the complex compared to the same mol. fr. in EtOH. Quantum calculations allowed to evaluate the effect of individual solvents on BA inclusion into β-CD, which is impossible to obtain by calorimetric or by NMR experiments. For molecular complexes of BA and β-CD the increase in the solvation of BA lead to a decrease of lg*K*^0^ values in water–ethanol mixtures. In water–DMSO solvent, nearly equal increase of solvation of both BA and β-CD results in a decrease of [BA⊂β-CD] complex stability. 

The data obtained in this work will be contributed to predict the thermodynamic parameters of the reactions of the formation of complexes of hydrophobic biologically active molecules with CD in various media. This research may help developing cyclodextrin-based pharmaceutical formulations through the modulation of the appropriate solvent mixture that would thus be employed industrially as a mean to control the reactions/processes carried out in liquid phases. 

## Figures and Tables

**Table 1:** The observed chemical shifts of β-CD protons for the [BA⊂β-CD] system in D_2_O at the different “host”–“guest” ratio (relative to the β-CD).

C_BA_/C_β-CD_	Δ, ppm					
	H1	H5	H6	H3	H2	H4
0.4	−0.017	−0.082	−0.014	−0.065	−0.019	−0.01
0.6	−0.025	−0.091	−0.027	−0.1	−0.028	−0.015
0.8	−0.028	−0.1071	−0.0273	−0.118	−0.031	−0.016
1	−0.032	−0.1245	−0.0307	−0.137	−0.037	−0.019
1.2	−0.038	−0.145	−0.027	−0.159	−0.044	−0.022
1.4	−0.04	−0.147	−0.029	−0.162	−0.045	−0.024

**Table 2:** Binding constants and complexation induced chemical shifts for [BA⊂β-CD] inclusion complex in D_2_O solution and in D_2_O-DMSO-*d*_6_ mixed solvent with different molar fractions *X*_(DMSO-*d*6)_.

*X*_(DMSO-*d*6)_, mol. fr.	K, M^−1^	Δ(H5), ppm	Δ(H3), ppm
0	388.5	−0.1986	−0.2147
0.05	368.6	−0.0900	−0.1409
0.075	157.5	−0.1013	−0.1537
0.10	30.8	−0.1068	−0.1963
***X*_(DMSO-*d*6)_, mol. fr.**	**K**	**Δ(H5)**	**Δ(H3)**
0	388.5	−0.1986	−0.2147
0.05	368.6	−0.0900	−0.1409
0.075	157.5	−0.1013	−0.1537
0.10	30.8	−0.1068	−0.1963

**Table 3:** The observed complexation induced chemical shifts for the [BA⊂β-CD] solution in D_2_O-EtOH at the *X*_(EtOH)_ = 0.075 mol. fr. and different “host”–“guest” ratio.

C_BA_/C_β-CD_	Δ(BA), ppm			Δ(β-CD), ppm				
	2,6	4	3,5	H1	5H	H6	H3	H2
0.2	0.0058	0.0604	0.0399	−0.0113	−0.0256	−0.0162	−0.0172	−0.0081
0.4	0.0118	0.0699	0.0396	−0.0145	−0.0421	−0.0232	−0.0269	−0.0089
0.6	0.0132	0.0591	0.0339	−0.0233	−0.0567	−0.0329	−0.0390	−0.0165
0.8	0.0122	0.0589	0.0354	−0.0246	−0.0664	−0.0332	−0.0479	−0.0138
1	0.0116	0.0514	0.0311	−0.0272	−0.0722	−0.0325	−0.0529	−0.0154
1.2	0.0056	0.0469	0.0318	−0.0267	−0.0779	−0.0386	−0.0552	−0.0091
1.4	0.0149	0.0460	0.0316	−0.0236	−0.0809	−0.0394	−0.0549	−0.0081
1.6	0.0101	0.0466	0.0293	−0.0258	−0.0837	−0.0419	−0.0562	−0.0120
1.8	0.0167	0.0426	0.0319	−0.0308	−0.0839	−0.0358	−0.0579	−0.0145
2	0.0096	0.0337	0.0219	−0.0304	−0.0883	−0.0381	−0.0629	−0.0141

**Table 4:** Binding constants and complexation-induced chemical shifts of β-CD for the [BA⊂β-CD] system in O-EtOH with the different molar fractions *X*_(C2H5OH)_.

*X*_(EtOH)_, mol. fr.	K, M^−1^	Δ(H5), ppm	Δ(H3), ppm
0	388.5	−0.1986	−0.2147
0.05	292.3	−0.1341	−0.1851
0.075	249.4	−0.0750	−0.1179
0.10	193.1	−0.1201	−0.1499
***X*_(EtOH)_, mol. fr.**	**K**	**Δ(H5), ppm**	**Δ(H3), ppm**
0	388.5	−0.1986	−0.2147
0.05	292.3	−0.1341	−0.1851
0.075	249.4	−0.0750	−0.1179
0.10	193.1	−0.1201	−0.1499

**Table 5:** The interaction energy (E_int_) corresponding to the formation of three [BA⊂β-CD] interaction configurations in different solvents.

Configuration	HF	PR	TF
in vacuum			
E_int_,kJ mol^−1^	−59.09	−60.20	−102.86
in water			
E_int_,kJ mol^−1^	−44.41	−38.27	−53.29
in methanol			
E_int_,kJ mol^−1^	−32.61	−25.44	−33.30
in DMSO			
E_int_,kJ mol^−1^	−2.54	−8.32	−19.09

**Table 6:** Geometrical and electronic properties of the isolated BA and the BA in the TF configurations.

Parameter	BA	BA in TF (in Vacuum)	BA in TF (in Water)	BA in TF (in Methanol)	BA in TF (in DMSO)
d(H1-O2), Å	0.969	0.988	0.977	0.974	0.974
BO(H1-O2)	0.868	0.803	0.798	0.824	0.818
d(O2-C4), Å	1.344	1.331	1.326	1.330	1.334
<H1O2C4C5, degree	180.00	175.96	178.70	178.89	176.97
<O3C4C5C6, degree	179.88	161.72	174.20	179.99	177.97
q(BA), e	0.000	−0.021	0.007	0.008	0.004

**Table 7:** Concentration conditions of experiments, distribution coefficients and changes in the Gibbs energy change of benzoic acid (∆*_tr_G*^0^(BA)) in the Hex-H_2_O and Hex-H_2_O-DMSO systems, T = 298.2 K.

*X*_DMSO_, mol. fr.	[BA]^H2O-DMSO^ × 10^4^, mol/L	[BA]^Hex^ × 10^5^, mol/L	K_1_	K_2_	Δ*_tr_G*^0^(BA), kJ/mol
0.0	4.108 4.058	6.860 7.078	0.17	-	0
0.05	5.422 5.380 5.421	4.289 4.666 4.470	-	0.08	−1.70
0.1	6.387 6.356 6.376	4.592 4.905 4.696	-	0.07	−2.07
0.15	5.320 5.271 5.280	5.478 5.975 5.875		0.11	−1.12
0.2	5.371 5.415 5.426	4.731 4.287 4.176	-	0.08	−1.91
0.3	5.410 5.316 5.529	5.452 6.393 4.262	-	0.10	−1.35

## Data Availability

The data presented in this study are available on request from the corresponding authors.

## Supplementary Materials

The following are available online. Table S1. Observed chemical shifts of β-CD protons for the [BA⊂β-CD] system in D_2_O. Table S2. Observed chemical shifts of β-CD protons for the [BA⊂β-CD] system in D_2_O (relative to β-CD). Table S3. Observed chemical shifts of β-CD protons for the [BA⊂β-CD] system in the solvent D_2_O-DMSO-*d*_6_ (*X*_(DMSO-*d*6)_ = 0.05 mol. fr.). Table S4. Observed chemical shifts of β-CD protons for the [BA⊂β-CD] system (relative to β-CD) in the solvent D_2_O-DMSO-*d*_6_ (*X*_(DMSO-*d*6)_ = 0.05 mol. fr.). Table S5. Observed chemical shifts of β-CD protons for the [BA⊂β-CD] system in the solvent D_2_O-DMSO-*d*_6_ (*X*_(DMSO-*d*6)_ = 0.075 mol. fr.). Table S6. Observed chemical shifts of β-CD protons for the [BA⊂β-CD] system (relative to β-CD) in the solvent D_2_O-DMSO-*d*_6_ (*X*_(DMSO-*d*6)_ = 0.075 mol. fr.). Table S7. Observed chemical shifts of β-CD protons for the [BA⊂β-CD] system in the solvent D_2_O-DMSO-*d*_6_ (*X*_(DMSO-*d*6)_ = 0.10 mol. fr.). Table S8. Observed chemical shifts of β-CD protons for the [BA⊂β-CD] system (relative to β-CD) in the solvent D_2_O-DMSO-*d*_6_ (*X*_(DMSO-*d*6)_ = 0.10 mol. fr.). SII. xyz coordinate files of the structures shown in Figure 11 and Figure 12.

## Sample Availability

Samples of the compounds are not available from the authors.
