# Supplementary material for: Complexation of Cyclodextrins with Benzoic Acid in Water-Organic Solvents: A Solvation-Thermodynamic Approach
Source: Molecules. 2021 Jul 21;26(15):4408. doi: 10.3390/molecules26154408 (PMC8348358; doi:10.3390/molecules26154408)
Supplement: Supplementary file 1 [file molecules-26-04408-s001.zip › molecules-1276959-supplementary.pdf]

Supplementary Materials

Table S1. Observed chemical shifts of β-CD protons for the [BA⊂β-CD] system in $D_2O$.

| C(G) / C(H) | δ, ppm | | | | | |
|---|---|---|---|---|---|---|
| | H1 | H5 | H6 | H3 | H2 | H4 |
| 0 | 4.941 | 3.837 | 3.75 | 3.726 | 3.521 | 3.455 |
| 0.4 | 4.924 | 3.755 | 3.736 | 3.661 | 3.502 | 3.445 |
| 0.6 | 4.916 | 3.746 | 3.723 | 3.626 | 3.493 | 3.44 |
| 0.8 | 4.913 | 3.7299 | 3.7227 | 3.608 | 3.49 | 3.439 |
| 1 | 4.909 | 3.7125 | 3.7193 | 3.589 | 3.484 | 3.436 |
| 1.2 | 4.903 | 3.692 | 3.723 | 3.567 | 3.477 | 3.433 |
| 1.4 | 4.901 | 3.69 | 3.721 | 3.564 | 3.476 | 3.431 |

Table S2.Observed chemical shifts of β-CD protons for the [BA⊂β-CD] system in $D_2O$ (relative to β-CD).

| C(G) / C(H) | Δ, ppm | | | | | |
|---|---|---|---|---|---|---|
| | H1 | H5 | H6 | H3 | H2 | H4 |
| 0.4 | -0.017 | -0.082 | -0.014 | -0.065 | -0.019 | -0.01 |
| 0.6 | -0.025 | -0.091 | -0.027 | -0.1 | -0.028 | -0.015 |
| 0.8 | -0.028 | -0.1071 | -0.0273 | -0.118 | -0.031 | -0.016 |
| 1 | -0.032 | -0.1245 | -0.0307 | -0.137 | -0.037 | -0.019 |
| 1.2 | -0.038 | -0.145 | -0.027 | -0.159 | -0.044 | -0.022 |
| 1.4 | -0.04 | -0.147 | -0.029 | -0.162 | -0.045 | -0.024 |

Table S3. Observed chemical shifts of β-CD protons for the [BA⊂β-CD] system in the solvent $D_2O$-DMSO-d6 ($X_{(DMSO\text{-}d6)}$=0.05).

| C(G) / C(H) | δ, ppm | | | | | |
|---|---|---|---|---|---|---|
| | H1 | H6 | H5 | H3 | H2 | H4 |
| 0 | 4.957 | 3.768 | 3.778 | 3.683 | 3.536 | 3.474 |
| 0.2 | 4.952 | 3.751 | 3.761 | 3.656 | 3.532 | 3.471 |
| 0.4 | 4.947 | 3.754 | 3.747 | 3.636 | 3.527 | 3.467 |
| 0.6 | 4.941 | 3.744 | 3.733 | 3.616 | 3.522 | 3.464 |
| 0.8 | 4.941 | 3.744 | 3.728 | 3.607 | 3.521 | 3.465 |
| 1 | 4.935 | 3.737 | 3.717 | 3.591 | 3.518 | 3.460 |
| 1.2 | 4.934 | 3.741 | 3.713 | 3.586 | 3.517 | 3.460 |
| 1.4 | 4.934 | 3.741 | 3.711 | 3.581 | 3.515 | 3.460 |
| 1.6 | 4.930 | 3.737 | 3.704 | 3.570 | 3.512 | 3.458 |
| 1.8 | 4.929 | 3.736 | 3.702 | 3.568 | 3.512 | 3.457 |
| 2 | 4.926 | 3.726 | 3.697 | 3.560 | 3.509 | 3.459 |

Table S4.Observed chemical shifts of β-CD protons for the [BA⊂β-CD] system (relative to β-CD) in the solvent $D_2O$-DMSO-d6 ($X_{(DMSO\text{-}d6)}$=0.05).

| C(G) / C(H) | Δ, ppm | | | | | |
|---|---|---|---|---|---|---|
| | H1 | H6 | H5 | H3 | H2 | H4 |
| 0.2 | -0.005 | -0.017 | -0.017 | -0.027 | -0.003 | -0.002 |
| 0.4 | -0.010 | -0.013 | -0.031 | -0.046 | -0.009 | -0.006 |
| 0.6 | -0.015 | -0.024 | -0.045 | -0.066 | -0.014 | -0.009 |
| 0.8 | -0.016 | -0.023 | -0.050 | -0.076 | -0.014 | -0.009 |
| 1 | -0.022 | -0.031 | -0.061 | -0.092 | -0.018 | -0.014 |
| 1.2 | -0.023 | -0.027 | -0.065 | -0.097 | -0.018 | -0.014 |
| 1.4 | -0.022 | -0.027 | -0.067 | -0.101 | -0.020 | -0.013 |
| 1.6 | -0.027 | -0.031 | -0.074 | -0.112 | -0.0235 | -0.016 |
| 1.8 | -0.0279 | -0.032 | -0.076 | -0.114 | -0.024 | -0.017 |
| 2 | -0.0314 | -0.042 | -0.081 | -0.123 | -0.027 | -0.015 |

Table S5. Observed chemical shifts of β-CD protons for the [BA⊂β-CD] system in the solvent $D_2O$-DMSO-d6 ($X_{(DMSO\text{-}d6)}$=0.075).

| C(G) / C(H) | δ, ppm | | | | | |
|---|---|---|---|---|---|---|
| | H1 | H5 | H6 | H3 | H2 | H4 |
| 0 | 4,9642 | 3,7779 | 3,7648 | 3,6896 | 3,4801 | 3,5403 |
| 0,2 | 4,9547 | 3,7674 | 3,7519 | 3,6677 | 3,4723 | 3,5338 |
| 0,4 | 4,9485 | 3,7561 | 3,7512 | 3,6514 | 3,4674 | 3,5271 |
| 0,6 | 4,9446 | 3,7451 | 3,7509 | 3,6373 | 3,4648 | 3,5218 |
| 0,8 | 4,9414 | 3,7383 | 3,7501 | 3,6237 | 3,4631 | 3,5209 |
| 1 | 4,9371 | 3,7274 | 3,7474 | 3,6103 | 3,4597 | 3,5177 |
| 1,2 | 4,9322 | 3,7221 | 3,7475 | 3,6043 | 3,4566 | 3,5141 |
| 1,4 | 4,9303 | 3,7141 | 3,7390 | 3,5928 | 3,4543 | 3,5115 |
| 1,6 | 4,9275 | 3,7038 | 3,7363 | 3,5850 | 3,4531 | 3,5093 |
| 1,8 | 4,9266 | 3,7035 | 3,7325 | 3,5786 | 3,4522 | 3,5079 |
| 2 | 4,9259 | 3,7024 | 3,7298 | 3,5771 | 3,4527 | 3,5069 |
| 2,5 | 4,9252 | 3,7021 | 3,7322 | 3,5734 | 3,4521 | 3,5076 |

Table S6.Observed chemical shifts of β-CD protons for the [BA⊂β-CD] system (relative to β-CD) in the solvent $D_2O$-DMSO-d6 ($X_{(DMSO\text{-}d6)}$=0.075).

| C(G) / C(H) | Δ, ppm | | | | | |
|---|---|---|---|---|---|---|
| | H1 | H5 | H6 | H3 | H2 | H4 |
| 0,2 | -0,0095 | -0,0105 | -0,0129 | -0,0220 | -0,0079 | -0,0065 |
| 0,4 | -0,0157 | -0,0219 | -0,0136 | -0,0382 | -0,0128 | -0,0132 |
| 0,6 | -0,0196 | -0,03299 | -0,0139 | -0,0523 | -0,0154 | -0,0185 |
| 0,8 | -0,0227 | -0,0396 | -0,0147 | -0,0659 | -0,0171 | -0,0194 |
| 1 | -0,0271 | -0,0505 | -0,0174 | -0,0794 | -0,0205 | -0,0226 |
| 1,2 | -0,0319 | -0,0558 | -0,0172 | -0,0853 | -0,0235 | -0,0262 |
| 1,4 | -0,0336 | -0,0639 | -0,0258 | -0,0969 | -0,0259 | -0,0287 |
| 1,6 | -0,0367 | -0,0742 | -0,0285 | -0,1047 | -0,0270 | -0,0309 |
| 1,8 | -0,0376 | -0,0745 | -0,0323 | -0,1110 | -0,0279 | -0,0323 |
| 2 | -0,0382 | -0,0755 | -0,0350 | -0,1126 | -0,0274 | -0,0333 |
| 2,5 | -0,0390 | -0,0758 | -0,0326 | -0,1162 | -0,0281 | -0,0327 |

Table S7. Observed chemical shifts of β-CD protonsfor the [BA⊂β-CD] system in the solvent $D_2O$-DMSO-d6 ($X_{(DMSO\text{-}d6)}$=0.10).

| C(BA)/C(β-CD) | δ, ppm | | | | | |
|---|---|---|---|---|---|---|
| | H1 | H6 | H5 | H3 | H2 | H4 |
| 0 | 4.969 | 3.781 | 3.757 | 3.693 | 3.544 | 3.485 |
| 0.4 | 4.968 | 3.770 | 3.747 | 3.677 | 3.542 | 3.483 |
| 0.6 | 4.968 | 3.769 | 3.745 | 3.671 | 3.544 | 3.485 |
| 0.8 | 4.963 | 3.774 | 3.736 | 3.657 | 3.538 | 3.481 |
| 1.0 | 4.962 | 3.773 | 3.734 | 3.652 | 3.538 | 3.482 |
| 1.2 | 4.961 | 3.773 | 3.730 | 3.644 | 3.538 | 3.481 |
| 1.4 | 4.959 | 3.770 | 3.724 | 3.637 | 3.535 | 3.480 |

Table S8.Observed chemical shifts of β-CD protons for the [BA⊂β-CD] system (relative to β-CD) in the solvent $D_2O$-DMSO-d6 ($X_{(DMSO\text{-}d6)}$=0.10).

| C(BA)/C(β-CD) | Δ, ppm | | | | | |
|---|---|---|---|---|---|---|
| | H1 | H6 | H5 | H3 | H2 | H4 |
| 0 | 0 | 0 | 0 | 0 | 0 | 0 |
| 0.4 | -0.001 | -0.010 | -0.010 | -0.016 | -0.002 | -0.0016 |
| 0.6 | -0.001 | -0.011 | -0.012 | -0.021 | -0.000 | 0.0003 |
| 0.8 | -0.006 | -0.006 | -0.021 | -0.035 | -0.006 | -0.0040 |
| 1.0 | -0.006 | -0.007 | -0.023 | -0.041 | -0.005 | -0.0023 |
| 1.2 | -0.008 | -0.008 | -0.027 | -0.049 | -0.006 | -0.0033 |
| 1.4 | -0.010 | -0.011 | -0.033 | -0.056 | -0.009 | -0.0051 |

SII. xyz coordinate files of the structures shown in Figures 8 and 9 (in the vacuum)

**β-CD**

```
C    15.44070186134040   18.64528985603408    5.49010584628289
C    16.24220851320264   17.33583700566636    5.38795315339300
C    16.73775864472669   16.94405506114052    6.78192343288776
C    17.56887777346838   18.09949411603824    7.34345987300720
C    16.75473289677683   19.40352985392702    7.31567573062334
C    17.63204217122161   20.59323440059304    7.71539592474804
O    15.42065489591320   16.34123915679382    4.83745921456574
O    17.48504596272231   15.75950918838937    6.66187943603474
O    17.93296214936810   17.76339946046045    8.67387993805353
O    16.23613852413700   19.66948014078327    6.02474314780440
O    18.82698862585315   20.56144198810426    6.97261123268335
C    19.27461624042752   17.90580343914268    9.04307979046671
C    19.75801114337023   16.57202034517171    9.63751962565238
C    18.97538692535561   16.28940266074585   10.92108004824308
C    19.16468209326350   17.46735225739220   11.88043202882151
C    18.78417944308239   18.78808310267700   11.19108266935982
C    19.15823212086265   19.98239398586536   12.07378933647641
O    19.56523677055445   15.55577482221094    8.68810193768406
O    19.45371580635577   15.08175979346597   11.45905010924718
O    18.33515326686251   17.23994952165055   13.01248607970693
O    19.46046711454873   18.95020621322288    9.95672270474200
O    20.48459259157875   19.83200795848564   12.51889447192993
C    18.91642377357774   17.31391408874542   14.27985619682291
```

| | | | |
|---|---|---|---|
| C | 18.53908909718401 | 16.03421717739858 | 15.05251398228721 |
| C | 17.02281008217647 | 16.01948752426547 | 15.25717477674441 |
| C | 16.60265568681701 | 17.29590028222279 | 15.99357777546797 |
| C | 17.11755940207112 | 18.53383256667648 | 15.23722466353982 |
| C | 16.87919544972487 | 19.80461768202354 | 16.05779783350785 |
| O | 18.96393245204020 | 14.91133211275295 | 14.32411444777270 |
| O | 16.61220232995598 | 14.85039652453635 | 15.92857624315428 |
| O | 15.18999566140288 | 17.29576437913246 | 16.07327516282425 |
| O | 18.51066028053394 | 18.44884809593785 | 14.99002855723509 |
| O | 17.32204830980781 | 19.59324531206133 | 17.37843464494180 |
| C | 14.59070144695936 | 17.59090108904347 | 17.30685073170103 |
| C | 13.59600598000083 | 16.44662903832623 | 17.61175010842739 |
| C | 12.49354290749333 | 16.49119628394478 | 16.54683189336630 |
| C | 11.83331679471102 | 17.88290961469207 | 16.57952647010404 |
| C | 12.88934731462931 | 18.98376701598009 | 16.38535412022372 |
| C | 12.29127797974482 | 20.37067647234324 | 16.66502129043374 |
| O | 14.25288226187499 | 15.21831992539999 | 17.67253098471779 |
| O | 11.53710518810494 | 15.47242167308911 | 16.69464311474201 |
| O | 10.88292034341522 | 17.90842809718983 | 15.53474951493121 |
| O | 13.96428148571319 | 18.83799799794316 | 17.31006412122157 |
| O | 11.89143112413841 | 20.48676493102903 | 18.00261220996220 |
| C | 9.59435806385902 | 18.39503808175935 | 15.80628228543965 |
| C | 8.59687006382898 | 17.34747229470640 | 15.27703907671087 |
| C | 8.71898390266635 | 17.29362360443788 | 13.74792682944375 |
| C | 8.48153158487857 | 18.69275704039884 | 13.17051073907525 |
| C | 9.46258172345872 | 19.68922847000704 | 13.81236729279208 |
| C | 9.12821314171216 | 21.13204040017217 | 13.41191357198382 |
| O | 8.81174184463133 | 16.09102503225907 | 15.85116411744522 |
| O | 7.77597967955729 | 16.40442191007098 | 13.19986570968917 |
| O | 8.67973020379679 | 18.62242545550190 | 11.77471285618818 |
| O | 9.37750590361204 | 19.64971208576532 | 15.23561224734345 |
| O | 7.86839366540003 | 21.51768068758625 | 13.88655250898072 |
| C | 7.70941984368906 | 19.18875819071516 | 10.92771243923445 |
| C | 7.31758511132883 | 18.11773838271061 | 9.89505404689779 |
| C | 8.55179824514885 | 17.81087780866232 | 9.03261641374560 |
| C | 9.02722402114608 | 19.10835884600350 | 8.36578734569093 |
| C | 9.30729132252023 | 20.16129821448618 | 9.45280691984607 |
| C | 9.61501127794053 | 21.53989841981427 | 8.85680676855197 |
| O | 6.81300898391307 | 16.95853780950058 | 10.49220823746778 |
| O | 8.24642415653594 | 16.86140148511165 | 8.04360178054867 |
| O | 10.18776783717773 | 18.81439425890828 | 7.62240178888756 |
| O | 8.17396321890042 | 20.34645518848686 | 10.30180892466482 |
| O | 9.97760738663206 | 22.46571695984935 | 9.84633012354992 |
| C | 10.29298627601526 | 19.31774043440257 | 6.30937924621455 |
| C | 10.67843230367650 | 18.13888282437218 | 5.38972007956151 |
| C | 12.08117734438521 | 17.65304865072016 | 5.79552452459176 |
| C | 13.04569442578195 | 18.85062169704682 | 5.75358261536086 |
| C | 12.52850669870208 | 20.03067080598303 | 6.58923173702859 |
| C | 13.39271157955348 | 21.27563713080746 | 6.37334979284606 |
| O | 9.69612914333057 | 17.15412945689745 | 5.43418996840490 |
| O | 12.49807910938718 | 16.65300759208494 | 4.91068117039069 |
| O | 14.29890313926654 | 18.40508842011388 | 6.25833738130669 |
| O | 11.19778979847410 | 20.37790624622312 | 6.24047047205293 |

| | | | |
|---|---|---|---|
| O | 13.54539800417312 | 21.50351158811820 | 4.99300721262987 |
| H | 15.17137789259423 | 18.99515954293846 | 4.48536237471795 |
| H | 17.11695706579800 | 17.51002096119397 | 4.74199420851701 |
| H | 15.86761156915786 | 16.78186623989531 | 7.43747770833889 |
| H | 18.46151285906519 | 18.23238339073397 | 6.72073088644117 |
| H | 15.92660972281016 | 19.30724094012784 | 8.03420385345873 |
| H | 17.84568634461343 | 20.52157700811962 | 8.78979110831838 |
| H | 17.07213447499311 | 21.51590192936675 | 7.51380817065423 |
| H | 15.84150487664801 | 15.49328311935834 | 5.03447889830790 |
| H | 18.11197333852642 | 15.70516096967637 | 7.40407015003810 |
| H | 19.36842441196546 | 21.31660614707644 | 7.22431307867548 |
| H | 19.89305454175778 | 18.17458459765232 | 8.17749545054190 |
| H | 20.82567574202155 | 16.66309428046548 | 9.88885351453142 |
| H | 17.90515777675326 | 16.20289522423255 | 10.67559433027829 |
| H | 20.21821007575805 | 17.52082250650220 | 12.17868238656796 |
| H | 17.69746497000777 | 18.78724511775192 | 11.01977406505867 |
| H | 18.46825873087678 | 20.01735611783485 | 12.92729449076327 |
| H | 19.63707980721919 | 14.71641351685054 | 9.16318953691783 |
| H | 19.23131418844365 | 15.04702492463565 | 12.40486165268206 |
| H | 20.74141584292980 | 20.62201389922270 | 13.00531731950455 |
| H | 20.00813890820898 | 17.40056829744636 | 14.21003446228716 |
| H | 19.04110012559180 | 16.06139072367638 | 16.03459789784085 |
| H | 16.52674983646717 | 15.98465287435601 | 14.28205080284594 |
| H | 17.04518236266802 | 17.30082290278985 | 16.99910480857917 |
| H | 16.57406066516452 | 18.60599180569324 | 14.28412224882354 |
| H | 15.80611700378107 | 20.03569011244892 | 16.04658255910211 |
| H | 17.43489341536667 | 20.62193560772503 | 15.58357290476575 |
| H | 18.38199823466245 | 14.17200828752869 | 14.55355161493541 |
| H | 16.98266527460548 | 14.84218223582517 | 16.82168269470848 |
| H | 17.31196752632554 | 20.43435377917312 | 17.84657961651374 |
| H | 15.34499426244271 | 17.65544776165661 | 18.10285091036917 |
| H | 13.15763541901650 | 16.60834425793870 | 18.60488312348000 |
| H | 12.93580687690191 | 16.34189210991584 | 15.55563370706724 |
| H | 11.33821726194835 | 18.03410736109895 | 17.55013252703373 |
| H | 13.26534467877215 | 18.92624199347276 | 15.35563785762458 |
| H | 11.39883436323294 | 20.51970845900718 | 16.05552995134530 |
| H | 14.71871598197231 | 15.06696601772835 | 16.83183616438268 |
| H | 11.38770374765742 | 15.29681625373666 | 17.63162802760866 |
| H | 12.65789758817356 | 20.27914899867318 | 18.55203914725123 |
| H | 9.45028130538258 | 18.54628363640250 | 16.88506789839939 |
| H | 7.57636547757453 | 17.64449395812688 | 15.54558434712845 |
| H | 9.73773874586139 | 16.97091658563777 | 13.48218996647156 |
| H | 7.45289358802528 | 19.00408676830217 | 13.39690398727263 |
| H | 10.48014368139801 | 19.43273356094928 | 13.48976575745750 |
| H | 9.91208969051040 | 21.79838950635865 | 13.79708077077237 |
| H | 9.09313576931630 | 21.20556009434691 | 12.32462187660164 |
| H | 9.76947034583387 | 15.90159905798562 | 15.86606247554012 |
| H | 7.72836401859482 | 15.63678687616707 | 13.78644395211194 |
| H | 7.86314058544517 | 21.37542375210935 | 14.84155686193373 |
| H | 6.82844124152421 | 19.50797742200585 | 11.50045948353380 |
| H | 6.52258683443539 | 18.51105297106997 | 9.24986076094128 |
| H | 9.35668250250917 | 17.43170986201306 | 9.68135979766447 |
| H | 8.23035111157382 | 19.47115938477901 | 7.70160364334632 |

| | | | |
|---|---|---|---|
| H | 10.16004956808292 | 19.81964470608515 | 10.05571938092928 |
| H | 8.72895746745990 | 21.88554672238356 | 8.30764290983135 |
| H | 10.45588717427487 | 21.46458184515533 | 8.16641070246610 |
| H | 7.30741299848054 | 16.77087137718526 | 11.30970392834489 |
| H | 7.62641704420865 | 16.23071553473889 | 8.43602689548093 |
| H | 9.23377401173578 | 22.54383551165338 | 10.45652307344592 |
| H | 9.33343753556133 | 19.73746604160269 | 5.97847088365762 |
| H | 10.72032966542920 | 18.48427794813156 | 4.35165681950070 |
| H | 12.04128629449217 | 17.26879375059639 | 6.82813956448067 |
| H | 13.14549308922672 | 19.18068498551345 | 4.71287346638394 |
| H | 12.56595920055838 | 19.74599044895342 | 7.65135551197224 |
| H | 14.36819687178712 | 21.10669887491269 | 6.84590288825373 |
| H | 9.49257103346982 | 16.94491502801462 | 6.36053706991939 |
| H | 13.46085315071780 | 16.54161911130404 | 4.99258155704795 |
| H | 14.12347311705395 | 22.26284354758868 | 4.86756770182484 |
| H | 13.03701399050227 | 21.13691690242748 | 16.41277968183382 |
| H | 19.04643025039701 | 20.89562113932857 | 11.47587748374866 |
| H | 12.89324423755538 | 22.12643410532230 | 6.85610588672074 |

**BA**

| | | | |
|---|---|---|---|
| C | -0.17694494340000 | 0.02294029870000 | 0.00008027800000 |
| C | 0.49519586050000 | -1.19678399820000 | 0.00079432410000 |
| C | 0.54537801520000 | 1.21472193700000 | -0.00064855300000 |
| C | 1.87732871050000 | -1.21763676260000 | 0.00078379570000 |
| C | 1.92633534360000 | 1.18557805520000 | -0.00081535300000 |
| C | 2.59258842040000 | -0.03023283260000 | -0.00009769660000 |
| H | -0.06990995370000 | -2.11561301570000 | 0.00159137140000 |
| H | 0.00294695180000 | 2.14799943790000 | -0.00099408480000 |
| H | 2.40066486380000 | -2.16230206750000 | 0.00142372330000 |
| H | 2.48615419450000 | 2.10890876590000 | -0.00136105090000 |
| H | 3.67252287330000 | -0.05270843300000 | -0.00023354890000 |
| C | -1.65362453200000 | 0.09579062100000 | 0.00011419810000 |
| O | -2.29131484490000 | 1.11982858230000 | 0.00146365500000 |
| O | -2.24998078900000 | -1.10845829160000 | -0.00155540030000 |
| H | -3.21283330080000 | -0.99312048530000 | -0.00148442140000 |

**HF configuration**

| | | | |
|---|---|---|---|
| C | 15.46400541597389 | 18.63048113781739 | 5.57566854618340 |
| C | 16.30140886367949 | 17.34191892113914 | 5.49151535583528 |
| C | 16.83995296361337 | 17.00612866206534 | 6.88397060791698 |
| C | 17.65367158061786 | 18.19766668837685 | 7.38759802524275 |
| C | 16.81370909465356 | 19.48473520946312 | 7.33815767662540 |
| C | 17.68836368854133 | 20.70210316923868 | 7.65218255602821 |
| O | 15.49869367417458 | 16.30804002685224 | 4.98838734491470 |
| O | 17.61676736613585 | 15.83735049903202 | 6.79078441810753 |
| O | 18.04830486158595 | 17.90450430874906 | 8.71797456468213 |
| O | 16.24142988268334 | 19.69373960171207 | 6.05909942649232 |
| O | 18.84936393151114 | 20.66118988519144 | 6.85624637521797 |
| C | 19.37161060827991 | 18.17364053882003 | 9.08803627578323 |
| C | 20.00448244471422 | 16.87207323809546 | 9.60537639069851 |
| C | 19.23799024394784 | 16.41537958425106 | 10.84707642524542 |

| | | | |
|---|---|---|---|
| C | 19.24924388713077 | 17.53887198114130 | 11.89457189028474 |
| C | 18.76195673500189 | 18.85625197683679 | 11.26930511444437 |
| C | 18.98764389777794 | 20.02821232071961 | 12.22864255319885 |
| O | 19.93870075621272 | 15.90488793235261 | 8.58853248590987 |
| O | 19.85329177896144 | 15.23748487116311 | 11.30742537890673 |
| O | 18.35277236195112 | 17.19678913348874 | 12.94616448313985 |
| O | 19.43962382479614 | 19.17144265505319 | 10.06571478763884 |
| O | 20.31726029116608 | 20.00350978363005 | 12.68551205593775 |
| C | 18.89106134012876 | 16.78928055201324 | 14.16676074906754 |
| C | 18.17926688621113 | 15.49463179881745 | 14.60445041745193 |
| C | 16.68772315041134 | 15.79388819715476 | 14.77513735994994 |
| C | 16.51402115305659 | 16.93574868392908 | 15.78450163382131 |
| C | 17.38907325923477 | 18.14278736578491 | 15.38823062658001 |
| C | 17.39037178176696 | 19.20367071787657 | 16.49282726890849 |
| O | 18.38946008192884 | 14.48591336160749 | 13.65017141910934 |
| O | 15.99312842149341 | 14.62704032393705 | 15.15325813079849 |
| O | 15.13855400725721 | 17.29432148936390 | 15.81954817030080 |
| O | 18.73623125387766 | 17.75990724746169 | 15.16463165681851 |
| O | 17.59227758258922 | 18.58561364428430 | 17.74559698326259 |
| C | 14.58195685399450 | 17.54626135414549 | 17.10225965424842 |
| C | 13.59944796854419 | 16.40409333413932 | 17.45729507667889 |
| C | 12.38496222090318 | 16.49518821272948 | 16.53103482101877 |
| C | 11.74977199671632 | 17.89195739404687 | 16.64579949736815 |
| C | 12.79569987353089 | 18.98811762640355 | 16.39151628741221 |
| C | 12.26281052007228 | 20.36743822546284 | 16.80191776386022 |
| O | 14.23273962791649 | 15.16109224820972 | 17.39420009835069 |
| O | 11.43436209071246 | 15.48800584231748 | 16.76674290071934 |
| O | 10.74769806918109 | 17.90872525855271 | 15.65450859674614 |
| O | 13.98308031371053 | 18.79635693829339 | 17.16674079653978 |
| O | 11.97868327475473 | 20.41775716185068 | 18.17468737203705 |
| C | 9.52593521156963 | 18.54135746945826 | 15.90601501747503 |
| C | 8.44368793059746 | 17.54877691960004 | 15.43693571725400 |
| C | 8.54922655890430 | 17.40592512378250 | 13.91265057731056 |
| C | 8.45045478627427 | 18.78316483107357 | 13.24859774799460 |
| C | 9.53745653828086 | 19.70156507243336 | 13.83354131969695 |
| C | 9.38403395761700 | 21.13997790754965 | 13.32657521169560 |
| O | 8.59154125439575 | 16.30823301035603 | 16.06918666412493 |
| O | 7.51990412552436 | 16.57957762750269 | 13.41825667137932 |
| O | 8.63973570620898 | 18.61459129007469 | 11.85765177458831 |
| O | 9.41828130916581 | 19.77227245976532 | 15.25708861688883 |
| O | 8.18605476852118 | 21.72034770354244 | 13.76543713366844 |
| C | 7.63932130593038 | 19.06358317255609 | 10.98170495639684 |
| C | 7.33779340336119 | 17.92762734144752 | 9.98962098885221 |
| C | 8.60835306890019 | 17.66800965277639 | 9.16401440354142 |
| C | 8.99237977673839 | 18.96034973871188 | 8.43364966952152 |
| C | 9.16395346077083 | 20.09048629193734 | 9.46334354774174 |
| C | 9.34874442203854 | 21.45497042474154 | 8.79163207362221 |
| O | 6.89552081244482 | 16.75916879086856 | 10.61699680500638 |
| O | 8.39262688683824 | 16.64529542699014 | 8.22537035992322 |
| O | 10.19416100738915 | 18.72462466659160 | 7.73509852265255 |
| O | 8.01781053096152 | 20.22518996779246 | 10.30284348922990 |
| O | 9.64484289031021 | 22.45878780635143 | 9.72792761849954 |
| C | 10.30676648162942 | 19.19522799202658 | 6.41138810070037 |

| | | | |
|---|---|---|---|
| C | 10.72672915047903 | 18.00221505879509 | 5.52528886453734 |
| C | 12.13775774569686 | 17.55916995994771 | 5.95146080730139 |
| C | 13.07121034190882 | 18.77910298092705 | 5.87704496009199 |
| C | 12.52712310588650 | 19.96315630669814 | 6.68979968080143 |
| C | 13.36681151368551 | 21.22038565001608 | 6.44683964278814 |
| O | 9.76652129558913 | 16.99737427358280 | 5.58955959164753 |
| O | 12.57921095585878 | 16.54503369792074 | 5.09497132945809 |
| O | 14.34445126197203 | 18.38382362127475 | 6.37445678227721 |
| O | 11.19044650584428 | 20.27361969952427 | 6.33008407392012 |
| O | 13.55336397669052 | 21.40371152766480 | 5.06444066854128 |
| H | 15.16388946336130 | 18.94166356335364 | 4.56675440135619 |
| H | 17.15532950462492 | 17.52369040473068 | 4.81991203396673 |
| H | 15.98976130728716 | 16.84290280531724 | 7.56474216769255 |
| H | 18.53178023575013 | 18.32623862569815 | 6.74316260115035 |
| H | 16.01724484410891 | 19.40679290471343 | 8.09445650831732 |
| H | 17.95527789021464 | 20.67172825832228 | 8.71701968818309 |
| H | 17.10315602985406 | 21.60618947593891 | 7.44117171888112 |
| H | 15.94462040943064 | 15.47815264164486 | 5.20605280850639 |
| H | 18.32251676872406 | 15.87374437895955 | 7.45961842628666 |
| H | 19.35098048018169 | 21.47111984722540 | 6.99574756488589 |
| H | 19.94728951730647 | 18.56029929022056 | 8.23821379967617 |
| H | 21.05037787277093 | 17.06861535247705 | 9.88352160069129 |
| H | 18.19226295783912 | 16.22210157958875 | 10.56269973625532 |
| H | 20.26613665537513 | 17.67764632321555 | 12.27720533893532 |
| H | 17.68696825505107 | 18.74748892166587 | 11.06585571016543 |
| H | 18.28811404078512 | 19.93406745529902 | 13.07070424871638 |
| H | 20.12658140067250 | 15.05269252878872 | 9.00619638471750 |
| H | 19.38436902478661 | 14.92797815441909 | 12.10038670066067 |
| H | 20.50189548306870 | 20.81927239899224 | 13.16179833405888 |
| H | 19.97243110474709 | 16.61905910117376 | 14.09657694110537 |
| H | 18.60149232517002 | 15.18500134376491 | 15.57586004640107 |
| H | 16.26721949291144 | 16.08935480737420 | 13.80856424364254 |
| H | 16.83770849762511 | 16.58571993156541 | 16.77288690969953 |
| H | 16.98212322101487 | 18.58136612481152 | 14.46814100701973 |
| H | 16.42517654213086 | 19.72853489672713 | 16.48117669560197 |
| H | 18.19490020367753 | 19.91097331168393 | 16.26948054153494 |
| H | 17.61087417272912 | 13.91017342257713 | 13.64604495417100 |
| H | 16.36044990632339 | 14.29286631024847 | 15.98385092316052 |
| H | 17.91172757013803 | 19.24323140322884 | 18.37109351470321 |
| H | 15.38613766039367 | 17.57977838705762 | 17.84825075033377 |
| H | 13.28306008564750 | 16.53414607837409 | 18.50029932999579 |
| H | 12.69906260971957 | 16.35627674504475 | 15.49026994880500 |
| H | 11.30893934712339 | 18.02746102573870 | 17.64566810651937 |
| H | 13.02041897266055 | 18.98107446879630 | 15.32146506378542 |
| H | 11.33008859383270 | 20.57278780944375 | 16.27507815119531 |
| H | 14.43857416541536 | 14.96970944483086 | 16.46346550166735 |
| H | 11.28456760234680 | 15.39678277872034 | 17.71587851537857 |
| H | 12.78881029511339 | 20.18248831302748 | 18.64392031540399 |
| H | 9.40580499777470 | 18.76692513651897 | 16.97536218094980 |
| H | 7.44861738546196 | 17.92202112235231 | 15.70236699549602 |
| H | 9.53139530008374 | 16.97509030172693 | 13.66453738402333 |
| H | 7.46148448180342 | 19.21555863655374 | 13.45112076755694 |
| H | 10.52147914907052 | 19.30034526250783 | 13.56241505987444 |

| | | | |
|---|---|---|---|
| H | 10.24845501174550 | 21.72962769846917 | 13.66280805689136 |
| H | 9.35029213437426 | 21.13935593491572 | 12.23691303363926 |
| H | 9.53180604330089 | 16.05164023717131 | 16.02949590401698 |
| H | 7.42835050731335 | 15.83621320355852 | 14.02977677558469 |
| H | 8.15052366141871 | 21.61277274097178 | 14.72422049638602 |
| H | 6.72966888571887 | 19.34060279105638 | 11.53169204338824 |
| H | 6.53640017800848 | 18.24793081884839 | 9.31122163980231 |
| H | 9.42792972411564 | 17.38843257351404 | 9.84346204478713 |
| H | 8.18652672080634 | 19.21686381215532 | 7.73166512490506 |
| H | 10.04390853561723 | 19.85372780508530 | 10.07744437266167 |
| H | 8.42904713470372 | 21.69884459320932 | 8.24346218476502 |
| H | 10.18421065370446 | 21.41240936556815 | 8.09064012943306 |
| H | 7.30312610370520 | 16.68577443453797 | 11.49841402073000 |
| H | 7.80668542861776 | 15.99819573795075 | 8.64303920756305 |
| H | 8.92215644467201 | 22.48242420042791 | 10.36819718998861 |
| H | 9.34385327996797 | 19.58736901988784 | 6.05705802894054 |
| H | 10.76693942936399 | 18.32053878808592 | 4.47849058249158 |
| H | 12.10210844517426 | 17.20303896163040 | 6.99414391158518 |
| H | 13.15021830471145 | 19.08944944230529 | 4.82826231799313 |
| H | 12.56942978391841 | 19.70578134393580 | 7.75889681086229 |
| H | 14.32953641370349 | 21.09229649279267 | 6.95584656384289 |
| H | 9.57947679151053 | 16.78866907196744 | 6.51990532832506 |
| H | 13.54483064222037 | 16.46180834840933 | 5.17518653274598 |
| H | 14.19258688870988 | 22.11025810864933 | 4.92795846830949 |
| H | 13.00752808776374 | 21.12736650291305 | 16.53005155228407 |
| H | 18.77693098966030 | 20.95678017936507 | 11.68340494489589 |
| H | 12.83139703070991 | 22.07515800356312 | 6.88187595490769 |
| C | 13.92170284459896 | 20.21359478131132 | 11.73143972594623 |
| C | 12.75593044743897 | 20.77869571413950 | 11.21651926904893 |
| C | 15.16378404601883 | 20.64932428371524 | 11.27621472575005 |
| C | 12.82759035473582 | 21.77710772942223 | 10.26432193335956 |
| C | 15.23003252135822 | 21.64289357802781 | 10.31814673288292 |
| C | 14.06725131082079 | 22.20841879451988 | 9.81693610564816 |
| H | 11.80427337534253 | 20.43116399056673 | 11.58863574271727 |
| H | 16.06348528439504 | 20.21069147119561 | 11.67831615180036 |
| H | 11.92102148006847 | 22.22507283643687 | 9.88900601273427 |
| H | 16.19250025699654 | 21.98473915619101 | 9.96804994221084 |
| H | 14.12808648422998 | 22.99672520094076 | 9.08196622940566 |
| C | 13.79668954465090 | 19.18806515026022 | 12.78552531560215 |
| O | 12.74197787125950 | 18.73453104449262 | 13.16866801757511 |
| O | 14.96112443191878 | 18.81922620860745 | 13.33435020497375 |
| H | 14.81789069578227 | 18.18406802501309 | 14.06618054970964 |

**PR configuration**

| | | | |
|---|---|---|---|
| C | 15.60245037491848 | 18.94903107872713 | 5.28480026370091 |
| C | 16.48585190281942 | 17.71355169550473 | 5.05391180657013 |
| C | 17.06438189377369 | 17.23716032696912 | 6.38733037112595 |
| C | 17.77786953568225 | 18.39521710812200 | 7.09964487042386 |
| C | 16.94471946298846 | 19.68960039895559 | 7.10741873152915 |
| C | 17.83890980065544 | 20.87830797819468 | 7.47519448958552 |
| O | 15.69886727159938 | 16.71398627185891 | 4.45838639975947 |
| O | 17.94990116597481 | 16.18266838855965 | 6.10430309303448 |
| O | 17.99288362394369 | 18.00113022205439 | 8.44579832315992 |

| | | | |
|---|---|---|---|
| O | 16.33288038597499 | 19.98885812523416 | 5.86248971207662 |
| O | 18.95159882251445 | 20.90901763588045 | 6.61396743704421 |
| C | 19.29440382086867 | 17.98172904122542 | 8.94509854662275 |
| C | 19.59676310488546 | 16.58316855497795 | 9.51459296603810 |
| C | 18.68740623429629 | 16.31989373478710 | 10.71799327230492 |
| C | 18.92714094523409 | 17.43552960315861 | 11.73778943062683 |
| C | 18.66664938758673 | 18.80164328837986 | 11.08187433256698 |
| C | 19.00545644916658 | 19.94565345982060 | 12.04080921238744 |
| O | 19.40842769531296 | 15.62241016473371 | 8.50856192083336 |
| O | 19.01941711410678 | 15.05232683480004 | 11.23689825632281 |
| O | 18.08255920949305 | 17.21181128433569 | 12.86220680313480 |
| O | 19.47449045817148 | 18.96685950404756 | 9.92795608089283 |
| O | 20.26550507658231 | 19.70978018586030 | 12.62120292054703 |
| C | 18.66550576467011 | 17.31417916416198 | 14.12852794232121 |
| C | 18.33390478563684 | 16.04235343401669 | 14.93846028425787 |
| C | 16.83946530128533 | 16.01722604859288 | 15.26839344413353 |
| C | 16.45331184817486 | 17.31644685470730 | 15.98651474969601 |
| C | 16.87538144552151 | 18.52275912353248 | 15.12894115493128 |
| C | 16.64274641808479 | 19.83775718744829 | 15.87833462872744 |
| O | 18.70633494564986 | 14.91727869234244 | 14.18679803426086 |
| O | 16.49844690192115 | 14.87474070552158 | 16.02010700849785 |
| O | 15.05385953239572 | 17.28036559792029 | 16.20711014929076 |
| O | 18.25738538248507 | 18.45888688191703 | 14.82212106294998 |
| O | 17.13489484078320 | 19.71775537105822 | 17.19260036878944 |
| C | 14.55510918971833 | 17.67972316908856 | 17.45559870628212 |
| C | 13.55652399439437 | 16.58950791217456 | 17.91138500074350 |
| C | 12.36295937999380 | 16.60262977125549 | 16.94724579869977 |
| C | 11.75465954760953 | 18.01914487552136 | 16.89465305459724 |
| C | 12.83015517336631 | 19.06825720306239 | 16.56624352943130 |
| C | 12.29830150967937 | 20.48989603856076 | 16.80348837768161 |
| O | 14.18496699392876 | 15.34642722229355 | 17.98509530669473 |
| O | 11.37572237203380 | 15.65292270061691 | 17.26080886397135 |
| O | 10.76235017584477 | 17.98099595398774 | 15.88503983856180 |
| O | 13.96033883254754 | 18.94174278218274 | 17.42537040272767 |
| O | 11.98913476678493 | 20.69284244206186 | 18.15486934240440 |
| C | 9.51037958727938 | 18.57000722145526 | 16.11572376416702 |
| C | 8.45349723394494 | 17.55999277406521 | 15.62953728316212 |
| C | 8.57631650095118 | 17.41636363501117 | 14.10464655174980 |
| C | 8.47062446158552 | 18.79056492935226 | 13.43234202252738 |
| C | 9.48591566055621 | 19.76626269524452 | 14.05332749793169 |
| C | 9.22824755807508 | 21.20149139789519 | 13.57520319284326 |
| O | 8.59421205164651 | 16.32347164120070 | 16.26799234792087 |
| O | 7.55944175725439 | 16.59007522093872 | 13.58975274189574 |
| O | 8.73362245273974 | 18.61077183986882 | 12.05247136296445 |
| O | 9.37303796634112 | 19.80267790423505 | 15.47517697128965 |
| O | 7.97397504466721 | 21.66513181984016 | 13.99107717809297 |
| C | 7.84441070432941 | 19.15308352731937 | 11.10569352536916 |
| C | 7.52739312561143 | 18.04525224109124 | 10.08535013610916 |
| C | 8.79563529864826 | 17.73652132924971 | 9.26910877699702 |
| C | 9.26417814925585 | 19.03108889167764 | 8.58957549484390 |
| C | 9.52260120293892 | 20.09075211341818 | 9.67592223742847 |
| C | 9.85252113185610 | 21.46141479522482 | 9.07206368689460 |
| O | 7.02357773807333 | 16.89020715452794 | 10.69217556517690 |

| | | | |
|---|---|---|---|
| O | 8.52991162670365 | 16.76373075725434 | 8.29214360186938 |
| O | 10.41978960847424 | 18.76130870018878 | 7.82422446414507 |
| O | 8.35886721579088 | 20.29021586210458 | 10.48214745960578 |
| O | 10.16967615784230 | 22.40565907385648 | 10.05865640898349 |
| C | 10.45670459790396 | 19.23423908979033 | 6.49236111031995 |
| C | 10.85128204714483 | 18.06299578657064 | 5.56493604129081 |
| C | 12.31489684862685 | 17.67542845586395 | 5.84907168111089 |
| C | 13.19067254405297 | 18.93186024105402 | 5.70950555811813 |
| C | 12.67543884359383 | 20.07706732217559 | 6.59539413787543 |
| C | 13.42988792734907 | 21.37876775749815 | 6.30988989378275 |
| O | 9.94669346850496 | 17.01480684100249 | 5.69337358675871 |
| O | 12.72042697617447 | 16.69138124190025 | 4.94075881718478 |
| O | 14.51404442460755 | 18.54680252429398 | 6.07878144301261 |
| O | 11.29910026029806 | 20.33690556107910 | 6.35569193127847 |
| O | 13.48474861118564 | 21.58985830381169 | 4.91878150214809 |
| H | 15.24765336468362 | 19.34550452384660 | 4.32582212916037 |
| H | 17.31892770805057 | 17.99657549783169 | 4.39308775573779 |
| H | 16.25496778005171 | 16.87749579791477 | 7.03866053473914 |
| H | 18.72755968683903 | 18.58857170014567 | 6.58647277454500 |
| H | 16.17387762613938 | 19.58864199292158 | 7.88604132643940 |
| H | 18.16083715656622 | 20.75311914156428 | 8.51823057693017 |
| H | 17.24718327553794 | 21.79794829194458 | 7.38132139147788 |
| H | 16.19816950965313 | 15.88924820692333 | 4.53264573066828 |
| H | 18.40861939236656 | 15.94161202182500 | 6.92926243781470 |
| H | 19.47238728784082 | 21.69526952184172 | 6.80681308431013 |
| H | 20.02244057837558 | 18.22694268696391 | 8.16036537592561 |
| H | 20.64423563770206 | 16.56589662521436 | 9.85650175195944 |
| H | 17.63916432287695 | 16.33427706122924 | 10.38277766587001 |
| H | 19.97876033777816 | 17.40538829048739 | 12.04920974077665 |
| H | 17.60473117538969 | 18.86260020391027 | 10.80157500020232 |
| H | 18.22807331420520 | 19.99492456107219 | 12.81391479952715 |
| H | 19.23291964424865 | 14.78484980121023 | 8.95917781460912 |
| H | 18.80268960014998 | 15.03625740794904 | 12.18252605107170 |
| H | 20.46893775299856 | 20.43178853559439 | 13.22457061602391 |
| H | 19.75500017272032 | 17.41735419173655 | 14.04500300607459 |
| H | 18.91045441431641 | 16.07961398753309 | 15.87940803013880 |
| H | 16.27466589950874 | 15.92700502100324 | 14.33606004911262 |
| H | 16.98681172349957 | 17.37744807866883 | 16.94509922435149 |
| H | 16.28473546814985 | 18.52172083173054 | 14.20138993717046 |
| H | 15.56710216146603 | 20.05336966937532 | 15.88921419145742 |
| H | 17.16760632072619 | 20.63242007705261 | 15.33452658702871 |
| H | 18.17323769844998 | 14.16674630006330 | 14.48571320821533 |
| H | 16.94696345773923 | 14.90333033580639 | 16.87582163228464 |
| H | 17.08108343710776 | 20.57764961812357 | 17.62196152169259 |
| H | 15.36900390089104 | 17.77302957983028 | 18.18744524230815 |
| H | 13.21479801030849 | 16.82193883734130 | 18.92800973933180 |
| H | 12.71353772646626 | 16.33790356287511 | 15.94452824458311 |
| H | 11.29978241515514 | 18.26394172122452 | 17.86561983102076 |
| H | 13.13435697130127 | 18.94059815755442 | 15.51889481503097 |
| H | 11.37481304520865 | 20.64139367724005 | 16.24320640900943 |
| H | 14.58874747716987 | 15.14565832763468 | 17.12402616608008 |
| H | 11.29081547589383 | 15.57067752859443 | 18.21814742187621 |
| H | 12.78178634024342 | 20.48664046206706 | 18.66642041932856 |

| | | | |
|---|---|---|---|
| H | 9.36951934804326 | 18.78718404840883 | 17.18360161138071 |
| H | 7.45197728989609 | 17.93082365695978 | 15.87603775463848 |
| H | 9.55948171555095 | 16.98146396881596 | 13.87139523965231 |
| H | 7.45570685773918 | 19.18283694081008 | 13.57882210114207 |
| H | 10.49766021953924 | 19.45210725545861 | 13.76651964433967 |
| H | 10.03080191092352 | 21.85063586289407 | 13.95034178177416 |
| H | 9.22754044955770 | 21.22203945416490 | 12.48515025354473 |
| H | 9.53993818856448 | 16.09363727076524 | 16.32244110663089 |
| H | 7.45239544480612 | 15.84620447675474 | 14.19804795487224 |
| H | 7.93758885475740 | 21.58193200954237 | 14.95222262158943 |
| H | 6.92193342647084 | 19.49219089600452 | 11.59612931442794 |
| H | 6.75256630518989 | 18.40587395530536 | 9.39668783974586 |
| H | 9.58355240810350 | 17.37523091904606 | 9.94648703062599 |
| H | 8.45554618226378 | 19.38390467632242 | 7.93400001349927 |
| H | 10.35590865710252 | 19.75947151665841 | 10.31041953479801 |
| H | 8.99284069251958 | 21.79493412876203 | 8.47580676789396 |
| H | 10.72371831614650 | 21.37458498998112 | 8.42229172092480 |
| H | 7.41251921988864 | 16.78605582572537 | 11.57786284817468 |
| H | 7.90991524076432 | 16.12975184583975 | 8.67875351114547 |
| H | 9.39588806120183 | 22.50528819332566 | 10.62698639832543 |
| H | 9.46441710526011 | 19.60008741276223 | 6.19495111892598 |
| H | 10.78710289385016 | 18.39678623604969 | 4.52347347359738 |
| H | 12.38634621089971 | 17.30178944400861 | 6.88414609552291 |
| H | 13.17330577259592 | 19.26116321471928 | 4.66460940811567 |
| H | 12.81571200472154 | 19.80113904245656 | 7.64997068521008 |
| H | 14.44204563225285 | 21.29731026229825 | 6.72531740039722 |
| H | 9.78778622483543 | 16.83452918552782 | 6.63428087273535 |
| H | 13.69141956101015 | 16.68190827464975 | 4.89261639795556 |
| H | 13.93930666357237 | 22.42129822485340 | 4.75039725440662 |
| H | 13.05249179408797 | 21.21238408500911 | 16.46268469695830 |
| H | 19.00927488533145 | 20.88136660721943 | 11.46670893447424 |
| H | 12.89432186730736 | 22.19686152685214 | 6.80859846337600 |
| C | 13.89533254761920 | 17.24682832456022 | 10.82054650573800 |
| C | 14.51783133534856 | 16.93512103696240 | 12.02380376481241 |
| C | 12.54001083776450 | 17.56136352543414 | 10.78500430556447 |
| C | 13.77800357618163 | 16.94867183282955 | 13.19230658372419 |
| C | 11.80626183097095 | 17.55456641267371 | 11.95500601757716 |
| C | 12.42612638675261 | 17.25196197276733 | 13.15625231597195 |
| H | 15.57384208893492 | 16.70548693198149 | 12.02898076716008 |
| H | 12.07204141594686 | 17.79852284011057 | 9.84099964410802 |
| H | 14.24765671261822 | 16.73021247063432 | 14.13828527283155 |
| H | 10.75422877891525 | 17.79010996780449 | 11.93366890758730 |
| H | 11.85239044359745 | 17.25831519936306 | 14.06965873988130 |
| C | 14.65377550109882 | 17.23085224109458 | 9.54625455921399 |
| O | 15.56886497412918 | 16.48667421916265 | 9.29649386798111 |
| O | 14.17526726936772 | 18.14517331524373 | 8.70796056729670 |
| H | 14.55223570614758 | 18.10320547129847 | 7.80159119707043 |

**TFconfiguration**

| | | | |
|---|---|---|---|
| C | 15.23436167103856 | 18.04170300010239 | 6.02448353324920 |
| C | 15.97117145870758 | 16.81478794640423 | 6.58096261865127 |
| C | 16.42183347639985 | 17.11060706300481 | 8.01890015503408 |

| | | | |
|---|---|---|---|
| C | 17.32039844688812 | 18.34961879859980 | 7.96866620955806 |
| C | 16.50429281813021 | 19.51940894337231 | 7.39019454215194 |
| C | 17.33992201040131 | 20.79401605876391 | 7.27589282816965 |
| O | 15.11396099945193 | 15.70420735684931 | 6.53265656027914 |
| O | 17.04933117412086 | 15.98254670720735 | 8.57156194050010 |
| O | 17.79903185255426 | 18.65305293053630 | 9.26642947192076 |
| O | 16.04479347446935 | 19.18873669131283 | 6.08138625362902 |
| O | 18.57596359612361 | 20.50271551610208 | 6.65767344945116 |
| C | 19.19121444700766 | 18.83282243340514 | 9.37985529856343 |
| C | 19.92325457643909 | 17.50376238037936 | 9.65752494450555 |
| C | 19.33199887186835 | 16.89529174686895 | 10.92743918418894 |
| C | 19.44586628094705 | 17.89753099210180 | 12.08148750882802 |
| C | 18.96946396997074 | 19.30602893909463 | 11.68430109629951 |
| C | 19.45187908216404 | 20.33927908483004 | 12.70719254500409 |
| O | 19.79600255940251 | 16.61336798928305 | 8.57203811909426 |
| O | 20.02885240111845 | 15.70313033122594 | 11.20717662295836 |
| O | 18.62318034806673 | 17.40175810365590 | 13.13252594617123 |
| O | 19.45737914099832 | 19.71998112915140 | 10.41869193479072 |
| O | 20.83257914206921 | 20.16758222956647 | 12.93098695016978 |
| C | 19.18730149941330 | 17.19402038973005 | 14.38882012332592 |
| C | 18.76297615734038 | 15.78824787265677 | 14.85860928487540 |
| C | 17.23795181440834 | 15.77427456610467 | 14.97282702513640 |
| C | 16.77450114960786 | 16.89272101570425 | 15.91241948868277 |
| C | 17.37914139731878 | 18.24617503096005 | 15.49877655993004 |
| C | 17.13527398995489 | 19.30744641766605 | 16.57583277602103 |
| O | 19.21594194828187 | 14.83933498503655 | 13.92656315524776 |
| O | 16.75646601196276 | 14.51340619582056 | 15.38265487745159 |
| O | 15.36059818676655 | 16.91027468159657 | 15.83899568263587 |
| O | 18.78259823765583 | 18.15727020762569 | 15.31890831543762 |
| O | 17.44530697200959 | 18.76807293327144 | 17.84184996939641 |
| C | 14.63465566677500 | 17.10296606016001 | 17.02380689106618 |
| C | 13.55147138579606 | 16.00489824517862 | 17.05196730156684 |
| C | 12.52238446211710 | 16.29815662908071 | 15.95218299021645 |
| C | 11.96224546771252 | 17.70815164378443 | 16.15430282731463 |
| C | 13.12179584450169 | 18.71792168465980 | 16.12634197678751 |
| C | 12.64006442350231 | 20.13189121768868 | 16.47875289268130 |
| O | 14.11777832774971 | 14.73538987679763 | 16.88758446794963 |
| O | 11.46439197353835 | 15.36966081961914 | 15.98939572156441 |
| O | 11.02566579785302 | 17.95554314344602 | 15.12904021173983 |
| O | 14.09010071471704 | 18.38356548646396 | 17.11984635529901 |
| O | 12.11869328866739 | 20.18301873598024 | 17.77816446804815 |
| C | 9.82030715000169 | 18.59103736925063 | 15.48160352621275 |
| C | 8.66192665930404 | 17.68616926245869 | 15.02045459050451 |
| C | 8.66198816459422 | 17.63874326691021 | 13.48638315629922 |
| C | 8.55543309512811 | 19.06616172584713 | 12.94222968021669 |
| C | 9.71354620089315 | 19.91151077264875 | 13.50200697368088 |
| C | 9.55346866800170 | 21.38874511395294 | 13.12304949420308 |
| O | 8.74510386515311 | 16.40211979190468 | 15.56692606194704 |
| O | 7.57964647955808 | 16.87525481706928 | 13.00920975503363 |
| O | 8.60808224039190 | 18.99866289434377 | 11.53307622698698 |
| O | 9.72739623252796 | 19.86810196933196 | 14.92791235860895 |
| O | 8.41486825037531 | 21.95443606175042 | 13.71091621827356 |
| C | 7.64686798465373 | 19.70472795760001 | 10.78787662525780 |

| | | | |
|---|---|---|---|
| C | 6.98042433129692 | 18.71295828190342 | 9.81835830181392 |
| C | 8.06392805315852 | 18.18068742794791 | 8.86938326080074 |
| C | 8.75722964881296 | 19.34207157072395 | 8.14364839337376 |
| C | 9.24328059047421 | 20.38232010006902 | 9.16929556605097 |
| C | 9.72230074645728 | 21.67981492706011 | 8.51143938193730 |
| O | 6.33341124667763 | 17.66707839364709 | 10.48389263876056 |
| O | 7.51702795440423 | 17.31052404421895 | 7.91152406194965 |
| O | 9.85704867063894 | 18.77820958320296 | 7.46031028904083 |
| O | 8.21875827861894 | 20.76740518696834 | 10.08544841884556 |
| O | 10.46297730215568 | 22.47663667155938 | 9.41147006964145 |
| C | 10.11275218294480 | 19.14806465579511 | 6.12870242639355 |
| C | 10.57438879162864 | 17.86067943449253 | 5.41350868183105 |
| C | 11.90109433849274 | 17.42373702177751 | 6.05909240692197 |
| C | 12.89621398920375 | 18.60103390153662 | 6.01592618095970 |
| C | 12.30550242727267 | 19.86896667944615 | 6.63951688434285 |
| C | 13.21378365962977 | 21.11183087281258 | 6.48151263508687 |
| O | 9.57050570416784 | 16.89735144268214 | 5.47299088257112 |
| O | 12.42079030156219 | 16.32451206610037 | 5.36343972958662 |
| O | 14.05553721814769 | 18.21118732577007 | 6.74569477806890 |
| O | 11.05108808396295 | 20.17944959075666 | 6.04123799683294 |
| O | 14.01391080358960 | 21.11992069719161 | 5.33299683141303 |
| H | 15.03096440404058 | 17.89160275871568 | 4.95492916346594 |
| H | 16.86164613025240 | 16.63062185740988 | 5.95715160459423 |
| H | 15.53546859727888 | 17.31614599288533 | 8.63169976319833 |
| H | 18.16229836373583 | 18.15247415620952 | 7.29427424880342 |
| H | 15.64778713467538 | 19.71737310290293 | 8.04816084264631 |
| H | 17.50276787356497 | 21.18948637670056 | 8.28716448707873 |
| H | 16.76780235120049 | 21.52040163201496 | 6.68984982724579 |
| H | 15.41008630710971 | 15.09685878089721 | 7.22591363052697 |
| H | 18.01081192759031 | 16.07427744055167 | 8.43658238136639 |
| H | 18.94801559446316 | 21.31898710495907 | 6.30981595507808 |
| H | 19.59344474596129 | 19.30142799335963 | 8.47200017252453 |
| H | 20.98365597643220 | 17.73897597866180 | 9.82849340547966 |
| H | 18.26718077808680 | 16.68169849676282 | 10.74945602758160 |
| H | 20.49315186382298 | 17.95427178562477 | 12.39954059244345 |
| H | 17.87166325742363 | 19.30065798166940 | 11.66850722442089 |
| H | 18.89544470938252 | 20.19600693000626 | 13.64402283759785 |
| H | 20.18021950506397 | 15.77639736859061 | 8.87046215086511 |
| H | 19.76489820565043 | 15.39078091587949 | 12.08794039048559 |
| H | 21.18141127514098 | 20.95811544317570 | 13.35418680212529 |
| H | 20.28163010553404 | 17.26738322063344 | 14.35626296967187 |
| H | 19.21215448590814 | 15.59655679998230 | 15.84743250265972 |
| H | 16.80479605149063 | 15.94210146572839 | 13.98282749539721 |
| H | 17.10303086371945 | 16.66804337026146 | 16.93702953779930 |
| H | 16.90195570401604 | 18.56041453343310 | 14.55900933678016 |
| H | 16.08251656150049 | 19.61485778808547 | 16.54119844619600 |
| H | 17.77689306474526 | 20.16559372838114 | 16.34767464506790 |
| H | 18.62039946509432 | 14.07730806487835 | 13.96372240313211 |
| H | 17.08551405045137 | 14.31427782478074 | 16.26947814650327 |
| H | 17.50204433823329 | 19.48758456507628 | 18.47865286831328 |
| H | 15.29145904546815 | 17.01318110237381 | 17.90020234742406 |
| H | 13.05018518711947 | 16.00028014904823 | 18.02621854561945 |
| H | 13.02483678350499 | 16.25790621219443 | 14.97396745312609 |

| | | | |
|---|---|---|---|
| H | 11.47181320280114 | 17.75893594965413 | 17.13644680060484 |
| H | 13.57942754624442 | 18.70656123080477 | 15.12811999119385 |
| H | 11.83627241820076 | 20.42829175100339 | 15.80355318548799 |
| H | 14.75569806185562 | 14.77359715333522 | 16.15248065764223 |
| H | 11.84290163922230 | 14.50582379382533 | 16.20388400746340 |
| H | 12.79433868523771 | 19.83645125636762 | 18.37459368645078 |
| H | 9.76544050509097 | 18.74909632588814 | 16.56693331763977 |
| H | 7.71077183840749 | 18.11132870664433 | 15.36319696645918 |
| H | 9.61055032574677 | 17.19861923231802 | 13.14197198229792 |
| H | 7.60223145155567 | 19.50386892964118 | 13.26818461942861 |
| H | 10.66144761361784 | 19.52697169763447 | 13.10676199862267 |
| H | 10.46023957907252 | 21.93150804709996 | 13.42229334755164 |
| H | 9.42435083970633 | 21.46651044946676 | 12.04322702635869 |
| H | 9.67125009553859 | 16.10039326275891 | 15.54899336398455 |
| H | 7.46201007367888 | 16.13393726543162 | 13.61978623610976 |
| H | 8.47223117152055 | 21.79423799934438 | 14.66144115609766 |
| H | 6.89579949774288 | 20.16288488709434 | 11.44448635220681 |
| H | 6.21461451840412 | 19.23738267111414 | 9.23474007229901 |
| H | 8.82346441855662 | 17.65180055373627 | 9.46714877143558 |
| H | 8.05200957292115 | 19.79672349315546 | 7.43557315708350 |
| H | 10.06699988025361 | 19.92188819325939 | 9.73042882698197 |
| H | 8.84678896406497 | 22.22956041446791 | 8.14806012707839 |
| H | 10.38208014386668 | 21.45907591462641 | 7.67276386859599 |
| H | 6.87980449241544 | 17.37208620152431 | 11.23430793039397 |
| H | 6.83285925868526 | 16.79132009034615 | 8.35659457713172 |
| H | 9.87069596382325 | 22.72876802435844 | 10.13139962215761 |
| H | 9.20457387547129 | 19.53776502637630 | 5.64854264389678 |
| H | 10.75051304765794 | 18.06289856198575 | 4.35261563929826 |
| H | 11.71347403020121 | 17.16622520948181 | 7.11306065849994 |
| H | 13.14536599052476 | 18.80257884833937 | 4.96637430621950 |
| H | 12.15227938601249 | 19.67341978136146 | 7.71028753484946 |
| H | 13.82393864160521 | 21.21257372863069 | 7.38544409247726 |
| H | 9.21268274010060 | 16.87414844972217 | 6.37429035821576 |
| H | 13.25911560222391 | 16.06389389553438 | 5.77879074981075 |
| H | 14.75366334848918 | 20.50365193873521 | 5.45871863903256 |
| H | 13.48214289851315 | 20.82921377756284 | 16.36861624653179 |
| H | 19.23785453492717 | 21.33377063206007 | 12.30162007073276 |
| H | 12.55561755640487 | 21.98040892097770 | 6.39681825766995 |
| C | 13.64543331961648 | 18.92937437320302 | 11.02497781930180 |
| C | 14.98320494576152 | 18.57262934913587 | 11.17464287301972 |
| C | 12.64512371054523 | 18.10476331444392 | 11.53463650546439 |
| C | 15.31848217928570 | 17.41919136709455 | 11.85931348283705 |
| C | 12.98690231472303 | 16.94486359635388 | 12.20450659608081 |
| C | 14.32103018184394 | 16.60713151503863 | 12.37682663688162 |
| H | 15.74564100248728 | 19.20321713579135 | 10.74229312373621 |
| H | 11.60853618384298 | 18.36717797152211 | 11.38662536417648 |
| H | 16.35851924454320 | 17.15852505214203 | 11.98415094184372 |
| H | 12.20975262398866 | 16.30222963652405 | 12.59102005384931 |
| H | 14.58213152280407 | 15.70023968056180 | 12.90348433726190 |
| C | 13.31341865051429 | 20.19366676907880 | 10.32275945525774 |
| O | 14.07692956954267 | 20.78434442282922 | 9.59428116607533 |
| O | 12.08589179456816 | 20.62437037291469 | 10.60187929694255 |
| H | 11.82380361382664 | 21.43071349420438 | 10.09494274492376 |
